# Targeted mutagenesis in mouse cells and embryos using an enhanced prime editor

**DOI:** 10.1186/s13059-021-02389-w

**Published:** 2021-06-03

**Authors:** Soo-Ji Park, Tae Yeong Jeong, Seung Kyun Shin, Da Eun Yoon, Soo-Yeon Lim, Sol Pin Kim, Jungmin Choi, Hyunji Lee, Jeong-Im Hong, Jinhee Ahn, Je Kyung Seong, Kyoungmi Kim

**Affiliations:** 1 Department of Physiology, Korea University College of Medicine, Seoul, 02841 Republic of Korea; 2 Department of Biomedical Sciences, Korea University College of Medicine, Seoul, 02841 Republic of Korea; 3 Korea Mouse Phenotyping Center, Seoul National University, Seoul, 08826 Republic of Korea; 4 Center for Genome Engineering, Institute for Basic Science, Daejeon, 34126 Republic of Korea; 5 Laboratory of Developmental Biology and Genomics, BK21 Program Plus for Advanced Veterinary Science, Research Institute for Veterinary Science, College of Veterinary Medicine, Seoul National University, Seoul, 08826 Republic of Korea; 6 Interdisciplinary Program for Bioinformatics, Program for Cancer Biology, BIO-MAX/N-Bio Institute, Seoul National University, Seoul, 08826 Republic of Korea

**Keywords:** Prime editor, *Igf2*, *Adamts20*, Mouse cells and embryos, Germline transmission, Dwarf phenotype, Proximal dead sgRNA, Chromatin-modulating peptides

## Abstract

Prime editors, novel genome-editing tools consisting of a CRISPR-Cas9 nickase and an engineered reverse transcriptase, can induce targeted mutagenesis. Nevertheless, much effort is required to optimize and improve the efficiency of prime-editing. Herein, we introduce two strategies to improve the editing efficiency using proximal dead sgRNA and chromatin-modulating peptides. We used enhanced prime-editing to generate *Igf2* mutant mice with editing frequencies of up to 47% and observed germline transmission, no off-target effects, and a dwarf phenotype. This improved prime-editing method can be efficiently applied to cell research and to generate mouse models.

**Supplementary Information:**

The online version contains supplementary material available at 10.1186/s13059-021-02389-w.

## Background

The CRISPR-Cas system has evolved with a variety of advanced genome-editing tools such as nucleases, base editors, and transposases, which can efficiently generate the desired target mutagenesis [1]. Especially, cytosine base editors (CBEs) and adenine base editors (ABEs) developed based on the CRISPR system can efficiently perform C•G to T•A and A•T to G•C substitutions, respectively [2, 3], in various organisms, including mice [4, 5]. Recently, a C to G base editor (CGBE1) that enables C to G base transversion in human cells has also been reported [6]. Nevertheless, precisely targeted mutagenesis involving one or more nucleotide insertions, conversions, or truncations is still challenging due to gene editing limitations resulting from the low efficiency of homology-directed repair (HDR).

Prime editor (PE), a new conceptual genome-editing tool, comprises a fusion protein with nickase Cas9 (H840A) and a commercial Moloney murine leukemia virus reverse transcriptase (M-MLV RT). PE is driven by a prime-editing guide RNA (pegRNA) that encodes the desired editing sequence [7]. This elaborate genome-editing system allows targeted mutagenesis of base-to-base conversion, as well as small insertions and deletions, without double-strand DNA breaks or donor DNA [7–10].

In this study, we improved the prime editor using proximal dead sgRNA (dsgRNA) and chromatin-modulating peptides (CMPs) at various target loci in mouse cell lines and embryos. We also created *Igf2* mutant mice using a PE with dsgRNA and identified germline transmission, no off-target effects, and a dwarf phenotype. We expect that these new improved prime-editing methods can be effectively applied to cell research and mouse model generation.

## Results and discussion

To verify the applicability of the prime-editing system in the mammalian genome, we used the tdTomato-expressing reporter system at the *AAVS1* locus in HEK293T cells. We designed the stop codon by inserting a T nucleotide into the tdTomato sequence using prime editor 3 (PE3) with a primer binding site (PBS) length of 8 nt and a reverse transcriptase (RT) template length of 17 nt (PBS8-RT17). Moreover, prime-editing guide RNA (pegRNA) was designed to remove the protospacer adjacent motif (PAM) sequence on the non-target strand to inhibit editing on the edited strand. We observed that tdTomato-negative cells represent 32% by flow cytometric analysis (Additional file 1: Figure S1), suggesting that the PE enables the desired target mutagenesis in our system.

Next, to apply the PE to mouse model generation, we tried to design targets involving eight transversion mutations or the insertion or deletion of one or more nucleotides. We first induced stop codons in two mouse genes, *Igf2* and *Adamts20*. *Igf2* can induce a dwarf phenotype caused by a mutation in the *Igf2* allele inherited from the father [11]. We inserted TA nucleotides into exon4 of the *Igf2* gene to generate a stop codon for loss of function. Besides, the nucleotide of the PAM sequence was changed from NGG (where “N” is any nucleotide base) to NCG to prevent continuous editing on the edited strand. *Adamts20* is a gene involved in the development of melanocytes. The premature stop codon at the E584 site of the *Adamts20* locus is linked to a typical white-belt phenotype [12]. We induced the conversion of nucleotides from CG to TT at exon12 of the *Adamts20* locus, resulting in a premature stop codon (E584*) and PAM modification (NGG to NAG) (Additional file 1: Figure S2a and S2b). To induce mutagenesis in the mouse targets, we used a PE3 system consisting of PE (nCas9 fused with engineered M-MLV RT), pegRNA, and nicking sgRNA (nsgRNA); the nsgRNA enhances the editing efficiency by promoting DNA repair activity through the cleavage of the non-edited strand [7].

First, we tested the editing efficiency of the pegRNAs with varying PBS lengths (8–14 nt) and RT template lengths (10–18 nt) to optimize the prime-editing at the *Igf2* and *Adamts20* sites. We excluded the length of PBS with thymine at the 3′-end (which could be part of the transcription termination signal) and the length of RT with cytosine at the 5′-end (which can interfere with the pegRNA structure) [7]. We transfected three plasmids encoding PE, pegRNA, and nsgRNA into NIH/3T3 cells via electroporation and harvested the cells after 72 h for targeted deep-sequencing. However, the editing efficiency with PE3 in the NIH/3T3 cells was less than 3% on the *Igf2* and *Adamts20* targets (Additional file 1: Figure S2c and 2d). These results suggest that the prime editor requires improvement for use in mouse model generation.

In one attempt to improve the editing efficiency of PE, we employed dsgRNAs based on the idea of proxy-CRISPR [13] instead of catalytically dead endonuclease to unwind the chromatin structure of the target sites. A dsgRNA is a 14- or 15-nt guide RNA that shows inactivated catalysis yet binds to the target site guiding Cas endonuclease [14]. Thus, we hypothesize that PE may play two roles: one is prime-editing at the target site with a pegRNA and the other is modulating the chromatin neighboring the target site with a dsgRNA. We designed proximal dsgRNAs adjacent to the *Igf2* and *Adamts20* target sites in the range of 7–62 nucleotide positions from the spacer of pegRNA. We applied proximal dsgRNAs to various pegRNA lengths at the *Igf2* and *Adamts20* sites to identify the editing efficiency, and interestingly, that of PE3 using proximal dsgRNA was improved in most groups. We chose PBS9-RT14 pegRNA and PBS11-RT13 pegRNA with the highest efficiencies for the *Igf2* and *Adamts20* targets, respectively (Additional file 1: Figure S2c and S2d).

Next, to select a dsgRNA with high editing efficiency, we designed and tested additional proximal dsgRNAs in *Igf2*, *Adamts20*, *Casp1* (4-bp deletion), *Hoxd13* (G to T conversion), *Angpt1* (CGG to TGA conversion), and *Ksr2* (TGAT insertion). The PE3 with a proximal dsgRNA was delivered into NIH/3T3 and C2C12 cells via electroporation with plasmids encoding PE, pegRNA, nsgRNA, and each proximal dsgRNA. Targeted deep-sequencing data reveals that proximal dsgRNA selectively improved the editing efficiency in most of the targets compared to PE3 (Additional file 1: Figure S3). Overall, the editing efficiency with dsgRNA depended on the proximal dsgRNA position, and so a screening process for an optimal dsgRNA is required for each target and cell type to induce effective targeted mutagenesis.

**Fig. 1:**
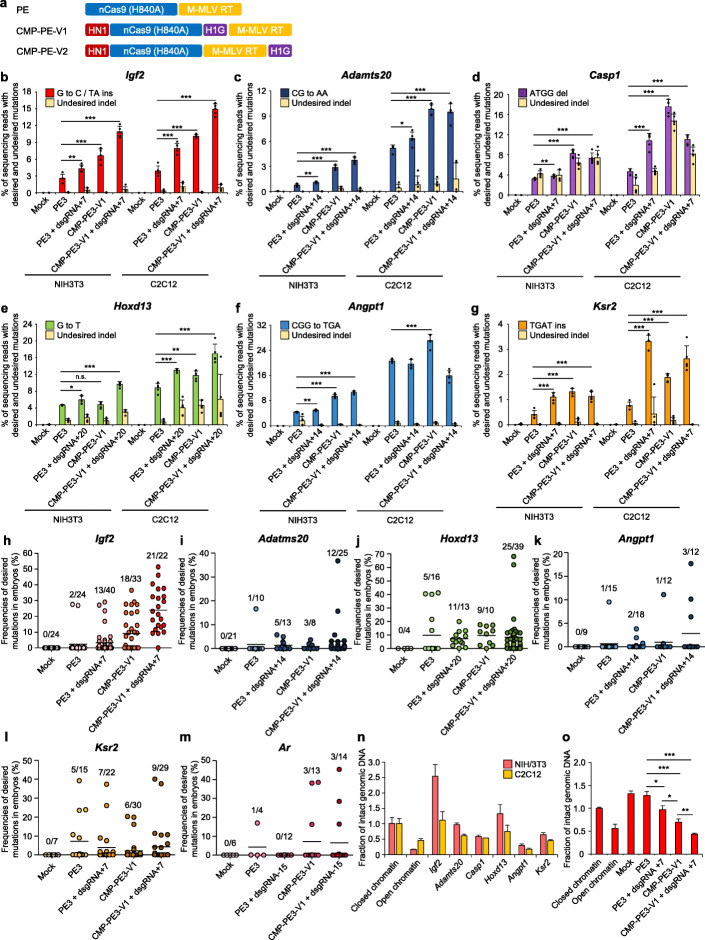
Improvement of prime-editing efficiency using chromatin-modulating peptides and proximal dsgRNA in mouse cells and embryos. **a** Schematic diagrams of the prime editor (PE), chromatin-modulating peptide-PE-Variant1 (CMP-PE-V1), and chromatin-modulating peptide-PE-Variant2 (CMP-PE-V2) constructs. PE consists of nCas9 (H840A) and M-MLV RT. CMP-PE-V1 has HN1 at the N-terminus of nCas9 and H1G at the C-terminus of nCas9 in the PE structure. CMP-PE-V2 has HN1 at the N-terminus of nCas9 and H1G at the C-terminus of M-MLV RT in the PE structure. HN1, high-mobility group nucleosome binding domain 1; H1G, histone H1 central globular domain. **b**–**g** Comparison of the prime-editing efficiencies of PE3, PE3+dsgRNA, CMP-PE3-V1, and CMP-PE3-V1+dsgRNA at the *Igf2* (**b**), *Adamts20* (**c**), *Casp1* (**d**), *Hoxd13* (**e**), *Angpt1* (**f**), and *Ksr2* (**g**) target sites in NIH/3T3 and C2C12 cells. Data and error bars show the mean ± standard deviation (s.d.) of five independent biological replicates (n = 5). *P*-values were obtained using two-tailed Student’s t-tests. *P <0.05, **P <0.01, ***P <0.001. **h**–**m** The components of the prime-editing system were injected into the pronucleus of the mouse zygote and analyzed 4 days after injection. The dots indicate the frequencies of the desired mutations in *Igf2* (**h**), *Adamts20* (**i**), *Hoxd13* (**j**), *Angpt1* (**k**), *Ksr2* (**l**), and *Ar* (**m**) from each blastocyst treated with PE3, PE3+dsgRNA, CMP-PE3-V1, or CMP-PE3-V1+dsgRNA. The numbers above the dots in the graph represent the number of edited embryos/total embryos. The black horizontal line denotes the mean of the frequencies of the desired mutations. **n** Fractions of intact genomic DNA from the *Igf2* and *Adamts20* target loci were measured using real-time qPCR after a DNase I digestion assay. The gene located at Chr 3: 71,026,628–71,026,685 (mouse genome build mm9) was used as the negative control (closed chromatin) and *Col6a1* was used as the positive control (open chromatin). Data and error bars show the mean ± s.d. of three independent experiments (n = 3). **o** After transfecting with plasmids encoding PE3, PE3+dsgRNA, CMP-PE3-V1, or CMP-PE3-V1+dsgRNA to C2C12, fractions of intact genomic DNA from the *Igf2* target locus were measured using real-time qPCR after a DNase I digestion assay. Data and error bars show the mean ± s.d. of three independent experiments (n = 3)

We also engineered the PE using chromatin-modulating peptides (CMPs), high-mobility group nucleosome binding domain 1 (HN1), and histone H1 central globular domain (H1G) [15] to increase the editing efficiency. CMP-PE-V1 consists of HN1 at the N-terminus and H1G at the C-terminus of nCas9. CMP-PE-V2 consists of HN1 at the N-terminus of nCas9 and H1G at the C-terminus of engineered M-MLV RT (Fig. 1a). We delivered engineered CMP-PE3-V1 (CMP-PE-V1 with pegRNA/nsgRNA) or CMP-PE3-V2 (CMP-PE-V2 with pegRNA/nsgRNA) into two mouse cell lines to compare the editing efficiency with PE3 (Additional file 1: Figure S4). We observed that CMP-PE3-V1 was significantly more efficient in all target sites compared to PE3. In particular, the editing efficiency of CMP-PE3-V1 was up to 2.55-fold higher in the *Igf2* target and up to 3.92-fold higher in the *Adamts20* target than by PE3, respectively, in NIH/3T3 (Additional file 1: Figure S4). These results suggest that engineered PEs using CMP HN1 and H1G can improve editing efficiency.

CMP-PE3-V1 and dsgRNA (CMP-PE3-V1 + dsgRNA) were delivered to mouse cells to test for any synergistic effects; CMP-PE3-V1+dsgRNA achieved improvements of up to 4.20-fold, 5.11-fold, and 3.56-fold prime-editing efficiency at the *Igf2*, *Adamts20*, and *Hoxd13* target sites, respectively, compared to PE3. However, the synergistic effect differed depending on the cell line and target (Fig. 1b–g). These results suggest that although CMP-PE3 could efficiently enhance the prime-editing in all of the targets and cell types, the efficiency of PE3+dsgRNA or CMP-PE3+dsgRNA varied depending on the target site and cell type.

Next, we carried out targeted mutagenesis in mouse embryos via microinjection using advanced prime-editing systems. We selected the *Igf2* target site that showed relatively low undesired mutations among the designed mouse targets. CMP-PE3-V1 and CMP-PE3-V1+dsgRNA appeared to have significantly higher editing efficiency at the *Igf2* target in mouse embryos. Notably, in the case of CMP-PE3-V1+dsgRNA, the desired mutations at the target site of the *Igf2* were observed in 21 out of 22 embryos (95%), suggesting that CMP-PE3-V1+dsgRNA has a much higher prime-editing efficiency than PE3 and PE3+dsgRNA (Fig. 1h and Additional file 1: Table S1). We further tested using CMP-PE-V1 and a proximal dsgRNA to verify the prime-editing efficiency at *Adamts20*, *Hoxd13*, *Angpt1*, *Ksr2*, and *Ar* in mouse embryos. Targeted deep-sequencing data revealed that CMP-PE-V1 and a proximal dsgRNA improved prime-editing efficiency in the *Adamts20*, *Hoxd13*, and *Angpt1* targets but not the *Ksr2* and *Ar* targets compared to PE3 (Fig. 1i–m and Additional file 1: Table S1). Taken together, our results suggest that improved prime-editing method using a proximal dsgRNA and chromatin-modulating peptides in mouse embryos can be efficient.

To determine whether CMP-PE-V1 and dsgRNA can improve chromatin accessibility by unraveling the chromatin structure of the target site, we performed a DNaseI digestion assay and qPCR to verify the chromatin status of each target site. In the NIH/3T3 cell line, *Igf2*, *Adamts20*, and *Hoxd13* appeared as relatively closed-chromatin structures, and in C2C12, all targets except *Igf2* were open chromatin (Fig. 1n and Additional file 1: Figure S5). This result suggests that even when the target sequences are the same for the two cell lines, their chromatin structure states are different. Furthermore, we analyzed whether CMP-PE-V1 or dsgRNA could alter the chromatin status at the *Igf2* target site (a representative target with a closed-chromatin structure). From the results, we identified that unlike PE3, the closed-chromatin structure was gradually opened by CMP-PE-V1, dsgRNA, or CMP-PE-V1+dsgRNA (Fig. 1o). These results are direct evidence that the use of CMP-PE-V1 or dsgRNA can improve prime-editing efficiency by unraveling chromatin structures and improving chromatin accessibility.

**Fig. 2:**
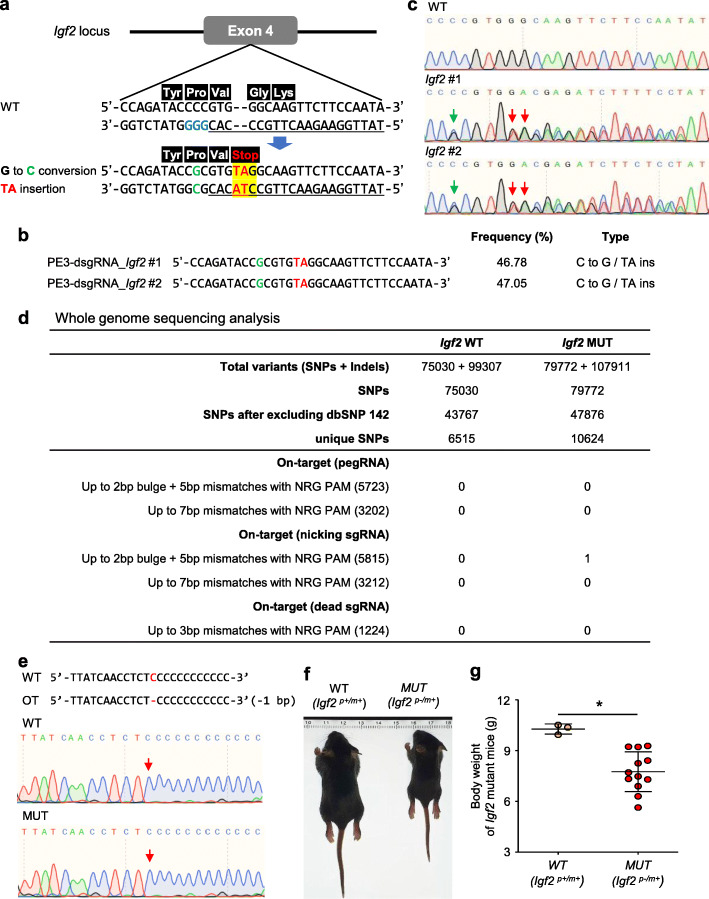
Targeted mutagenesis in mice using a prime editor 3 with proximal dsgRNA. **a** A schematic of the mutagenesis design at exon 4 in *Igf2*. **b** The genotypes of two mice harboring G to C conversion and TA insertion mutations induced by PE3 with proximal dsgRNA +7 in *Igf2*. **c** Sanger-sequencing chromatograms of the two mice harboring the desired mutations. The green arrow denotes the G to C conversion for modification of the PAM sequence, while the red arrow denotes the TA insertion for the stop codon. **d** Whole-genome sequencing analysis of the *Igf2* wild-type (WT) and mutant #1 mice. The potential off-target (OT) sites of pegRNA, nsgRNA, and dsgRNA were obtained using Cas-OFFinder. **e** Sequence alignment and Sanger-sequencing chromatogram of potential OT sites. **f** An image of the offspring after mating an *Igf2* p+/m− male (F1) with an *Igf2* p+/m+ female: wild-type (left) and *Igf2* mutant (right). **g** Bodyweight 2 weeks after birth. WT, wild-type; MUT, mutant; p, paternal allele; m, maternal allele. Error bars show the mean ± standard deviation (s.d.)

Next, we carried out targeted mutagenesis of *Igf2* in mouse embryos via microinjection using PBS9-RT14 and dsgRNA +7 with a relatively low frequency of undesired mutations. Injected mouse embryos were transplanted into surrogate mothers, and pups were obtained with G to C transversion and TA insertion at the *Igf2* locus at prime-editing frequencies of up to 47% (2 out of 10 pups) (Fig. 2a–c). Moreover, we identified that 7 out of 9 pups from an F1 littermate of the *Igf2* mutant mouse had germline transmission (Additional file 1: Figure S6).

To assess the off-target effects of the prime editor, we used Cas-OFFinder [16] to identify potential off-target sites by pegRNA and nsgRNA of the *Igf2* target with up to three nucleotide mismatches in the mouse genome. Off-target mutations were not detected at the potential off-target sites compared to the wild-type (Additional file 1: Figure S7). Moreover, we carried out whole-genome sequencing to identify off-target effects in the *Igf2* mutant mouse and found a single off-target site of nsgRNA. However, we determined that this site was a false positive through Sanger sequencing with genomic DNA (Fig. 2d, e). To identify the phenotype of the *Igf2* mutant mice, the *Igf2* p+/m− male (F1) was mated with a wild-type female. The *Igf2* p−/m+ mouse carrying the mutation for the *Igf2* gene inherited from the paternal allele exhibited the dwarf phenotype, which is consistent with the desired mutant genotype (Fig. 2f, g). These results suggest that the newly improved prime-editing tools can be effectively applied to mouse model generation.

## Conclusions

The prime editor system has not yet been tested to generate novel mouse models with the insertion or conversion of one or more nucleotides in the mouse genome. In this study, we introduced an improved prime editor using proximal dsgRNA and CMPs in mouse cells and embryos for efficient mutagenesis in mice. We demonstrated that the prime editor using proximal dsgRNA or CMPs can improve editing efficiency by opening the chromatin structure of the target site in mouse cell lines and embryos. However, simultaneous treatment of CMP-PE3 and proximal dsgRNA was more effective for targets with a closed-chromatin structure. We also obtained targeted mutant mice harboring a G to C conversion and a TA insertion simultaneously at the *Igf2* locus at frequencies of up to 47% using an improved prime-editing method and identified germline transmission, no off-target effects, and the dwarf phenotype. Our results suggest that the enhanced prime-editing method can be efficiently applied to the desired targets for mutagenesis, such as insertion or transversion of one or more nucleotides.

## Methods

### Plasmid DNA construction

Additional file 1: Table S2 and S4 lists the sequences of target-specific sgRNAs and primers for targeted deep sequencing. Using NEBuilder® HiFi DNA Assembly Master Mix (E2621L, NEB), HN1 and H1G oligos were fused to both sides of nCas9 in pCMV-PE2 (#132775, Addgene) to construct the chromatin-modulating peptide prime editor, according to the manufacturer’s protocol. To prepare the pegRNA expression vectors for specific mutagenesis on the *Igf2*, *Adamts20*, *Casp1*, and *Hoxd13* genes, pU6-pegRNA-GG-acceptor vector (#132777, Addgene) is used to insert a spacer, prime binding site, and reverse transcriptase template oligos at the BsaI enzyme cut site. nsgRNA and dsgRNA expression vectors are inserted into the pRG2-GG vector (#104174, Addgene).

### Lipofection and electroporation

tdTomato-expressing reporter system at the AAVS1 locus in HEK293T cells, NIH/3T3 (ATCC, CRL-1658), and C2C12 (ATCC, CRL-1772) were maintained in Dulbecco’s modified Eagle’s medium (DMEM; LM001-05, Welgene) supplemented with 10% FBS (S 001-01, Welgene) or BCS (26170-043, Gibco) in 5% CO_2_ and 37 °C. We transfected 2 × 10^4^ cells with three plasmids of 0.5 μg pegRNA, 2.15 μg PE2, 0.22 μg nsgRNA, and 1 μl Lipofectamine 2000 reagent (11668019, Thermo Fisher Scientific) in 50 μl Opti-MEM (31985070, Gibco), according to the manufacturer’s protocol. We cultured cells for 11 days for analysis after transfection. The number of NIH/3T3 cells and C2C12 cells used 1 × 10^5^ cells for electroporation. Each cell line was mixed with plasmids of 3 μg PE2 or CMP-PE, 0.7 μg pegRNA, 0.3 μg nsgRNA, and 0.25 μg dsgRNA and was transfected using the Neon™ Transfection System (MPK1096, Thermo Fisher Scientific), according to the manufacturer’s protocol. Transfected cells were harvested 72 h post-transfection and analyzed by targeted deep sequencing.

### Flow cytometric analysis

We analyzed the editing efficiency by PE3 in the tdTomato expression reporter HEK293T cell line 10 days post-transfection. We evaluated editing efficiency by gating tdTomato-negative cells using flow cytometry analysis (BD FACSCanto™, BD Biosciences). Additional file 1: Fig. S1 shows the results of sample gating.

### Preparation of DNA amplicon

Genomic DNA from tdTomato expression reporter HEK293T, NIH/3T3, C2C12 cells, and mouse embryos after transfection was extracted using DNeasy Blood & Tissue Kits (69506, Qiagen). We used Phusion™ High-Fidelity DNA Polymerase (F-530XL, Thermo Fisher Scientific) and Sungen (SG-PT02, Sun genetics) to amplify edited target sequences.

### In vitro transcription

Transcripts of PE2 and CMP-PE were produced using the mMESSAGE mMACHINE T7 Ultra Kit (AM1345, Invitrogen) and were purified using the MEGAclear™ Transcription Clean-Up Kit (AM1908, Invitrogen). According to the manufacturer’s protocol, the transcription of pegRNA, nsgRNA, and dsgRNA was induced using T7 RNA polymerase (M0251, NEB), and the RNAs were purified using ExpinTM CleanUp SV (113-150, GeneAll). The RNAs were quantified using NanoDrop One UV-Vis (Thermo Fisher Scientific).

### Animals

Our experiments were approved by the Institutional Animal Care and Use Committee (IACUC) of Seoul National University. C57BL/6N and ICR mice were used as embryo donations, and surrogate mothers were bred in a specific pathogen-free laboratory.

### Microinjection

HyperOva (KYD-010-EX-x5, CARD) and hCG (CG10-1vl, Sigma) were injected into C57BL/6N female mice at 48-h intervals to induce ovarian hyperstimulation. Following the injection of the hCG hormone, the female mice were mated with wild-type C57BL/6N male mice. Fertilized one-cell stage embryos were obtained from ampulla and incubated in M2 media (M7167, Sigma) until two pronuclei appeared. The prime-editing mixture for microinjection was prepared with a concentration of 100 ng for PE2 or CMP-PE mRNA, 65 ng for pegRNA, 32.5 ng for nsgRNA, and 22 ng for dsgRNA in 100 μl of Tris-EDTA (pH 7.4). Following microinjection, the embryos were cultured in KSOM media (MR-121-D, Millipore) for 4 days in a 37 °C incubator for blastocyst development. A portion of the two-cell stage embryos was transplanted into the oviducts of the pseudopregnant foster mothers.

### Genotyping and targeted deep sequencing

The targets were amplified by nested PCR using the primers listed in Additional file 1: Table S4. Libraries consisting of PCR amplicons were sequenced using the iSeq™ 100 Sequencing System (Illumina, Inc.). Sequencing data were analyzed by the CRISPR REGN Tools program (http://www.rgenome.net/) and the EUN program (https://daeunyoon.com/).

#### Statistics

Data were presented as the mean and standard deviation, and three or five independent biological replicates were performed. *P*-values were calculated using unpaired and two-sided Student’s t-tests.

### Whole-genome sequencing and variant calling

Genomic DNA was isolated from a mouse (C57BL/6N) ear using the DNeasy Blood & Tissue kit (69506, Qiagen). According to the manufacturer’s instructions, genomic DNA was sheared using a Covaris S2 ultra sonicator system and subjected to paired-end DNA library preparation with Truseq Nano DNA sample prep kit. Deep coverage (30x) whole-genome sequencing was carried out by 101 base paired-end sequencing on an Illumina Novaseq 6000 platform (Illumina). Sequence reads were aligned to the mouse reference genome GRCm38/mm10 using BWA-MEM [17]. Single nucleotide variants and small indels were called using GATK4 HaplotypeCaller [18], and the known variants present in dbSNP for mouse v142 (dbSNP142) were annotated with ANNOVAR [19]. The novel variants not present in dbSNP142 were further analyzed to identify their location at off-target sites. The putative off-target sites were compared with the candidates from Cas-OFFinder considering mismatch up to 7-bp or 2-bp bulge + 5-bp mismatches. All variants were manually confirmed by visualizing read plots.

### DNase I digestion assay and qPCR

We performed DNase I digestion assay following the previous studies [20–22]. After detaching the cells, we repeated washing the cells with cold 1XPBS and spin down at 900rpm for 5min twice. Cold RSB buffer (10mM Tris-HCl, 10mM NaCl and 3mM MgCl_2_) + 0.1% IGEPAL CA-630 (I8896, Sigma) wash was used to lyse the cells and we spin down at 500*g* for 10min at 4deg to pellet nuclei. After removing the supernatant, nuclei are incubated with or without DNase I (2-16U) for 20–30min at 37°C. Fifty millimolar EDTA was added to stop the reaction, and DNase I-digested DNA was purified using the DNeasy Blood & Tissue kit (69506, Qiagen). Real-time qPCR was performed using KAPA SYBR FAST qPCR Master Mix Kit (KR0389, Kapa Biosystems), and the fraction of intact genomic DNA was measured using the comparative C_T_ method [23].

## Supplementary Information

**Additional file 1: Supplementary figures and tables. Figure S1.** FACS analysis to validate the prime-editing system. **Figure S2.** Optimization of the prime-editing efficiency using various lengths of pegRNAs and proximal dsgRNA at the *Igf2* and *Adamts20* target sites. **Figure S3.** Improvement of prime-editing efficiency with proximal dsgRNAs in mouse cell lines. **Figure S4.** Improvement of prime-editing efficiency with chromatin-modulating peptides in mouse cell lines. **Figure S5.** Relative fractions of intact genomic DNA from closed chromatin and open chromatin regions in NIH/3T3 and C2C12 cells. **Figure S6.** Generation of F1 mice via germline transmission from *Igf2* mutant mice. **Figure S7.** Indel frequencies at the potential off-target sites of pegRNA and nsgRNA used for targeted mutagenesis of the *Igf2* target site. **Table S1.** Targeted mutagenesis in mouse embryos. **Table S2.** Sequences of pegRNAs, nsgRNAs, and dsgRNAs used in this study. **Table S3.** Sequencies of the on-target and the potential off-target sites of pegRNA and nsgRNA used for targeted mutagenesis of the *Igf2* target site. **Table S4.** Primer sequences used to amplify the target DNA in this study. **Table S5.** Primer sequences used for real-time qPCR. **Table S6.** Amino acid sequences of CMP-PE-V1 and CMP-PE-V2.

**Additional file 2.** Review history.

## Data Availability

The whole-genome sequencing and deep-sequencing data are available on the NCBI Sequence Read Archive (SRA) under accession number SRP320275 [24].
