# Supplementary material for: Targeted mutagenesis in mouse cells and embryos using an enhanced prime editor
Source: Genome Biol. 2021 Jun 3;22:170. doi: 10.1186/s13059-021-02389-w (PMC8173820; doi:10.1186/s13059-021-02389-w)
Supplement: Supplementary file 1 — Additional file 1: Supplementary figures and tables. Figure S1. FACS analysis to validate the prime-editing system. Figure S2. Optimization of the prime-editing efficiency using various lengths of pegRNAs and proximal dsgRNA at the Igf2 and Adamts20 target sites. Figure S3. Improvement of prime-editing efficiency with proximal dsgRNAs in mouse cell lines. Figure S4. Improvement of prime-editing efficiency with chromatin-modulating peptides in mouse cell lines. Figure S5. Relative fractions of intact genomic DNA from closed chromatin and open chromatin regions in NIH/3T3 and C2C12 cells. Figure S6. Generation of F1 mice via germline transmission from Igf2 mutant mice. Figure S7. Indel frequencies at the potential off-target sites of pegRNA and nsgRNA used for targeted mutagenesis of the Igf2 target site. Table S1. Targeted mutagenesis in mouse embryos. Table S2. Sequences of pegRNAs, nsgRNAs, and dsgRNAs used in this study. Table S3. Sequencies of the on-target and the potential off-target sites of pegRNA and nsgRNA used for targeted mutagenesis of the Igf2 target site. Table S4. Primer sequences used to amplify the target DNA in this study. Table S5. Primer sequences used for real-time qPCR. Table S6. Amino acid sequences of CMP-PE-V1 and CMP-PE-V2. [file 13059_2021_2389_MOESM1_ESM.docx]

**Supplementary Information:**

**Targeted mutagenesis in mouse cells and embryos using an enhanced prime editor**

**
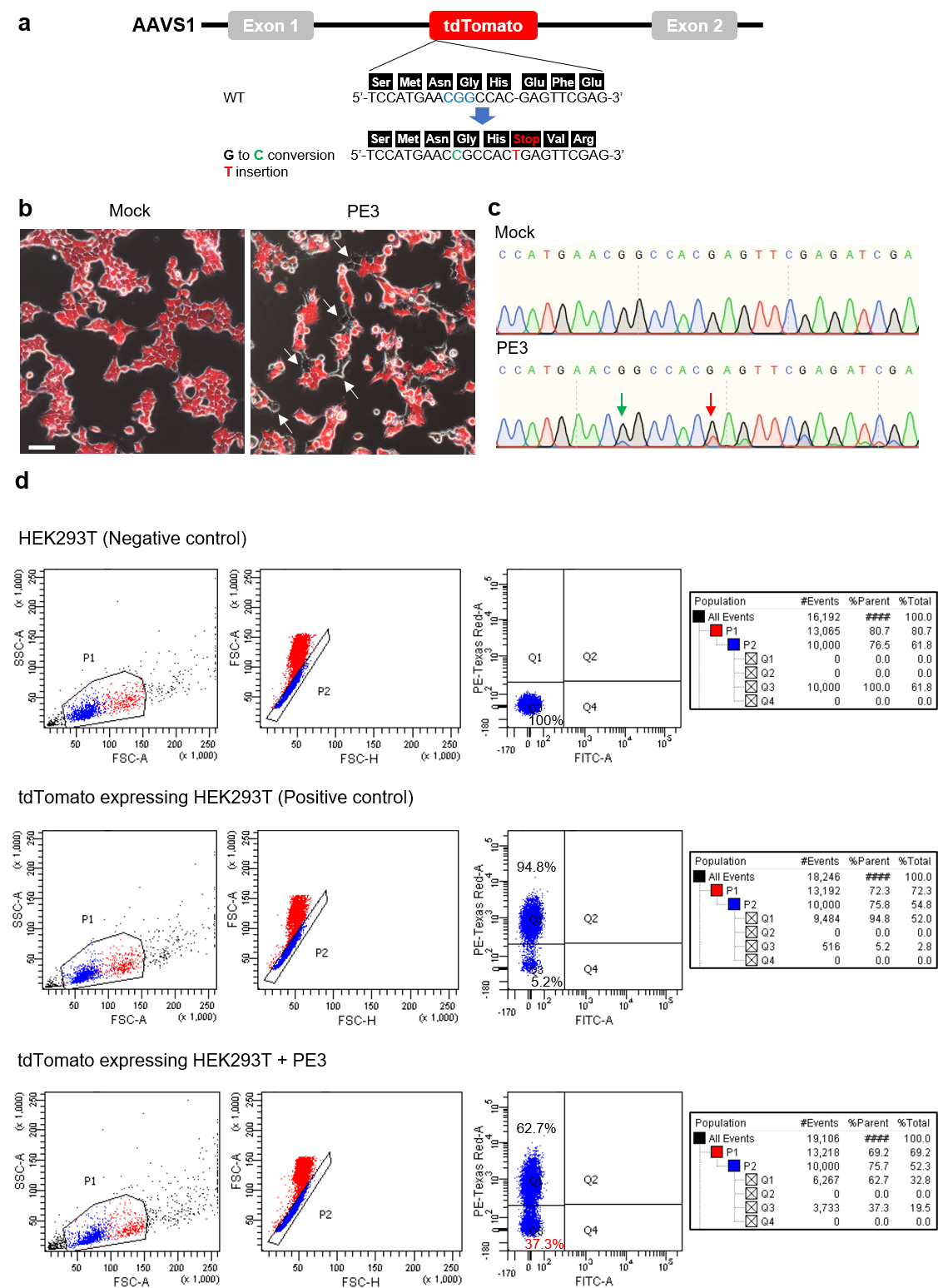
Supplementary Figures**

**Figure S1. FACS analysis to validate the prime-editing system.** The HEK293T cell line expressing tdTomato is used to test the activity of PE3. **a** Design of mutagenesis on the tdTomato gene located in *AAVS1* locus. **b** Images for PE3 efficiency in the tdTomato expression reporter HEK293T cell line. White scale bar, 100 μm; white arrow, tdTomato-negative cells. **c** Sanger sequencing chromatogram of mutants induced by PE3 in the tdTomato expression reporter HEK293T cell line. Blue arrow, G to C conversion for PAM modification; red arrow, T insertion for a stop codon. **d** Using FACS, tdTomato-negative and tdTomato-positive cells were isolated 11 days post-transfection with or without PE3. The cell populations revealed that PE3 efficiently induces the desired mutations on tdTomato, turning off the fluorescence expression.


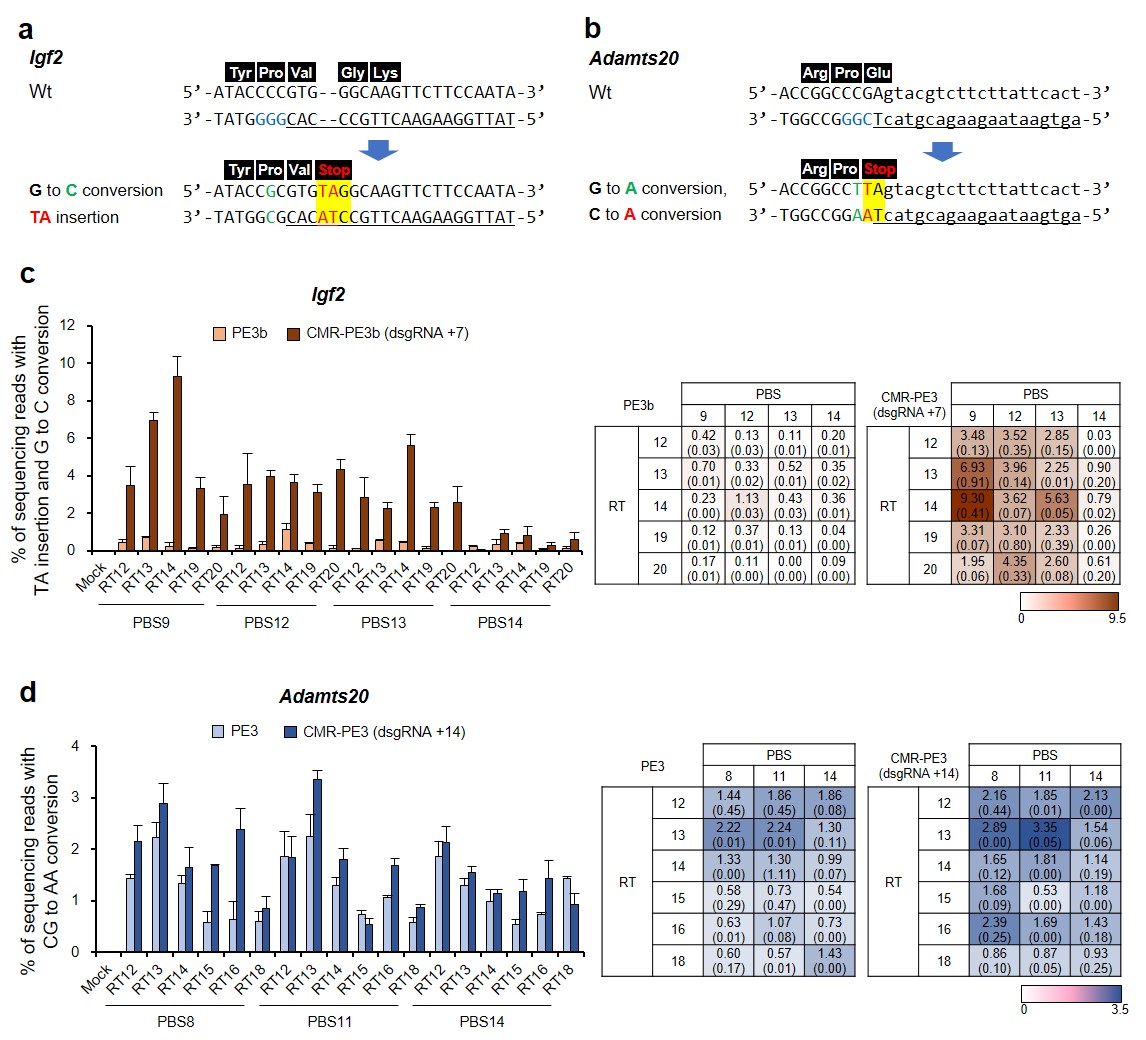


**Figure S2. Optimization of the prime-editing efficiency using various lengths of pegRNAs and proximal dsgRNA at the *Igf2* and *Adamts20* target sites.** **a, b** Schematic of the mutagenesis design at the *Igf2* (a) and *Adamts20* (b) target. **c, d** Comparison of the prime-editing efficiencies of PE3 and CMR-PE3 with diverse combinations of PBS lengths and RT template lengths in NIH/3T3 cells. Undesired indel frequencies are presented in parentheses. Data and error bars show the mean ± s.d. of three independent biological replicates ( n = 3 ).

**
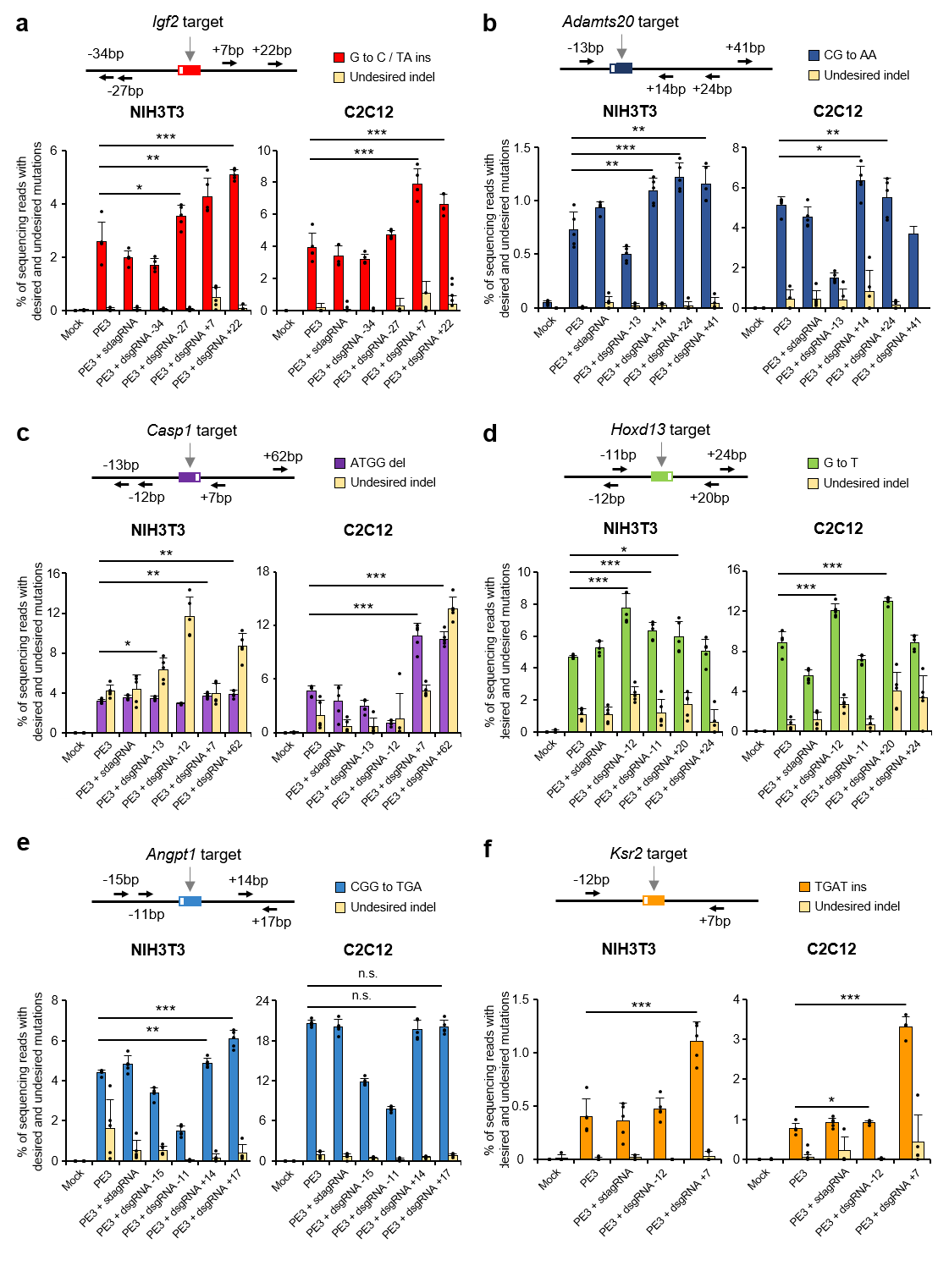
**

**Figure S3. Improvement of prime-editing efficiency with proximal dsgRNAs in mouse cell lines.** Comparison of the prime-editing efficiencies between PE3 and PE3 with proximal dsgRNAs at the *Igf2* (G to C conversion and TA insertion, **a**), *Adamts20* (CG to AA conversion, **b**), *Casp1* (ATGG deletion, **c**), *Hoxd13* (G to T conversion, **d**), *Angpt1* (CGG to TGA conversion, **e**), and *Ksr2* (TGAT insertion, **f**) target sites in NIH/3T3 and C2C12 cells. dsgRNAs were designed to locate in the range of 7-62 nt distant from the spacer of pegRNA. PE3+sdsgRNA represents the treatment of PE3 and scrambled dsgRNA (sdsgRNA). The sdsgRNA is four different dsgRNAs that do not bind to the target region. The white box in the target site indicates PAM for spacer of pegRNA, and the head of black arrow indicates PAM for dsgRNA. Data and error bars show the mean ± s.d. of five independent biological replicates (n = 5). *P*-values were obtained using two-tailed Student’s t-tests. n.s. not significant, **P* <0.05, ***P* <0.01, ****P* <0.001.

**
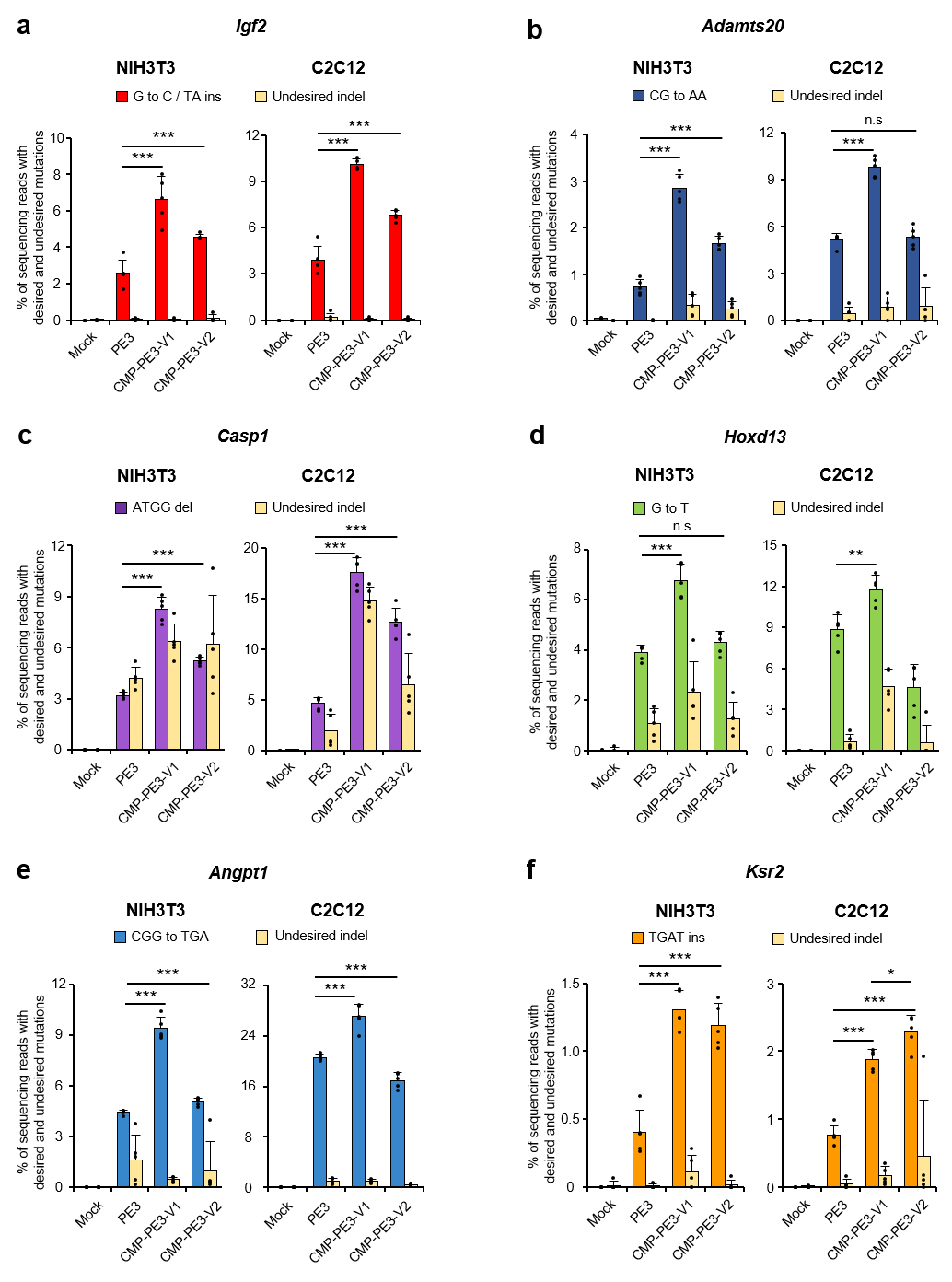
Figure S4. Improvement of prime-editing efficiency with chromatin-modulating peptides in mouse cell lines.** The prime-editing efficiencies of PE3, CMP-PE3-V1, and CMP-PE3-V2 at the *Igf2* (**a**), *Adamts20* (**b**), *Casp1* (**c**), *Hoxd13* (**d**), *Angpt1* (**e**), and *Ksr2* (**f**) target sites in NIH/3T3 and C2C12 cells. Data and error bars show the mean ± s.d. of five independent biological replicates (n = 5). *P*-values were obtained using two-tailed Student’s t-tests. n.s. not significant, **P* <0.05, ***P* <0.01, ****P* <0.001.

**
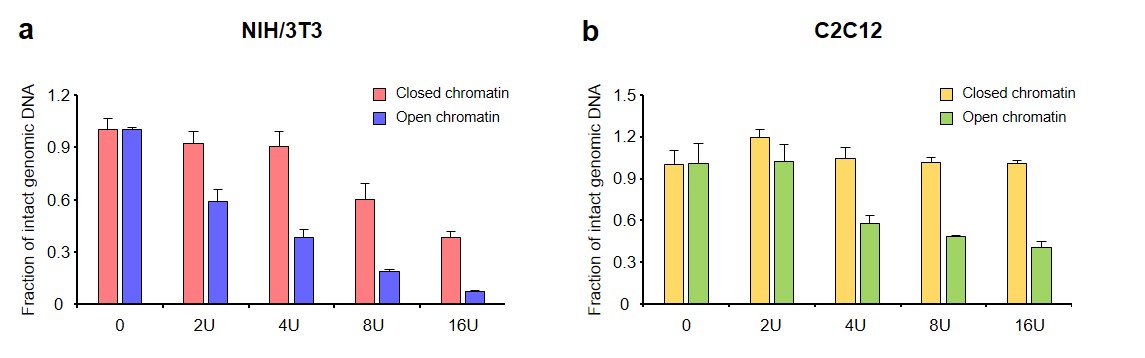
**

**Figure S5. Relative fractions of intact genomic DNA from closed chromatin and open chromatin regions in NIH/3T3 and C2C12 cells.** Nuclei of NIH/3T3 (**a**) and C2C12 (**b**) were treated with DNase I (2-16U), and fractions of intact DNA from closed chromatin and open chromatin region were calculated using the comparative C_T_ method. Data and error bars show the mean ± s.d. of three independent experiments ( n = 3 ).

**
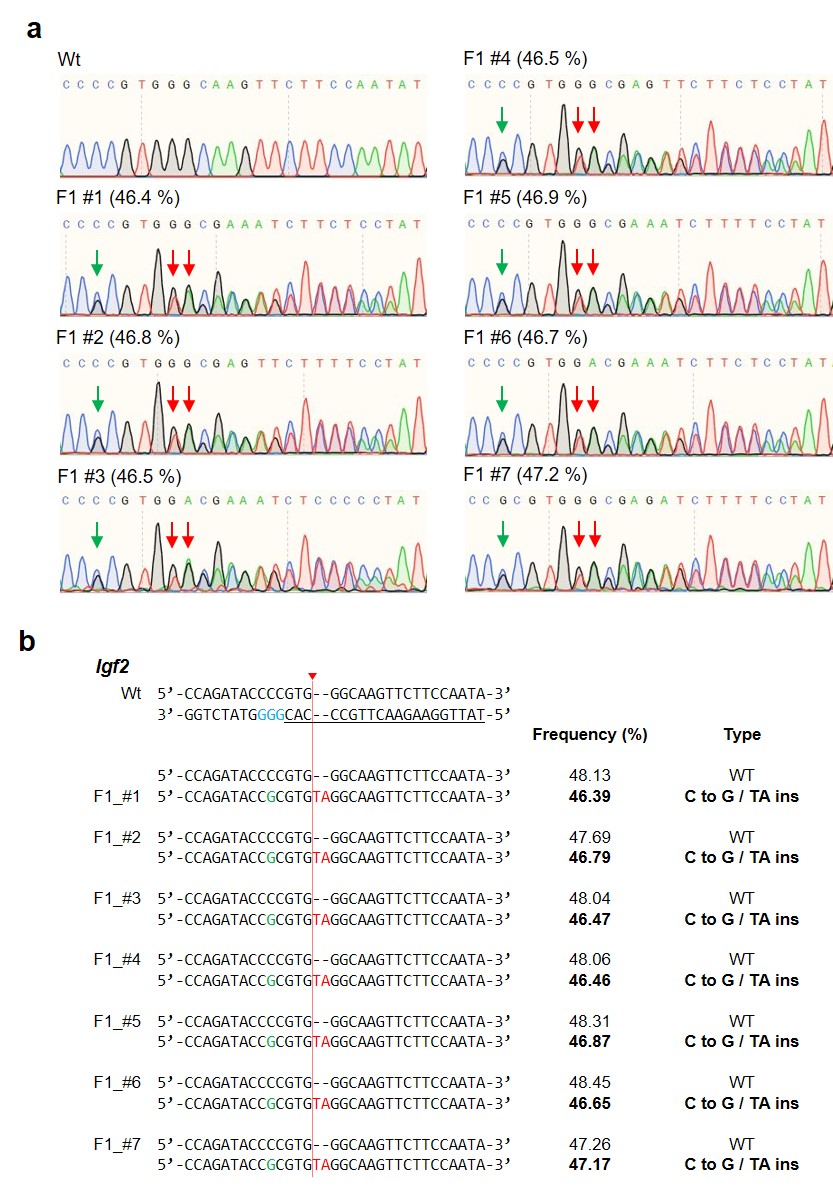
**

**Figure S6. Generation of F1 mice via germline transmission from *Igf2* mutant mice. a** Sanger sequencing chromatogram of the *Igf2* target site from F1 *Igf2* mutant mice. Green arrow, G to C conversion for PAM -free edit; red arrow, TA insertion for a stop codon. **b** Genotypes of F1 *Igf2* mutant mice harboring G to C conversion and TA insertion. Blue letters, PAM; red letters, desired edit; green letters, PAM-free edit.

**
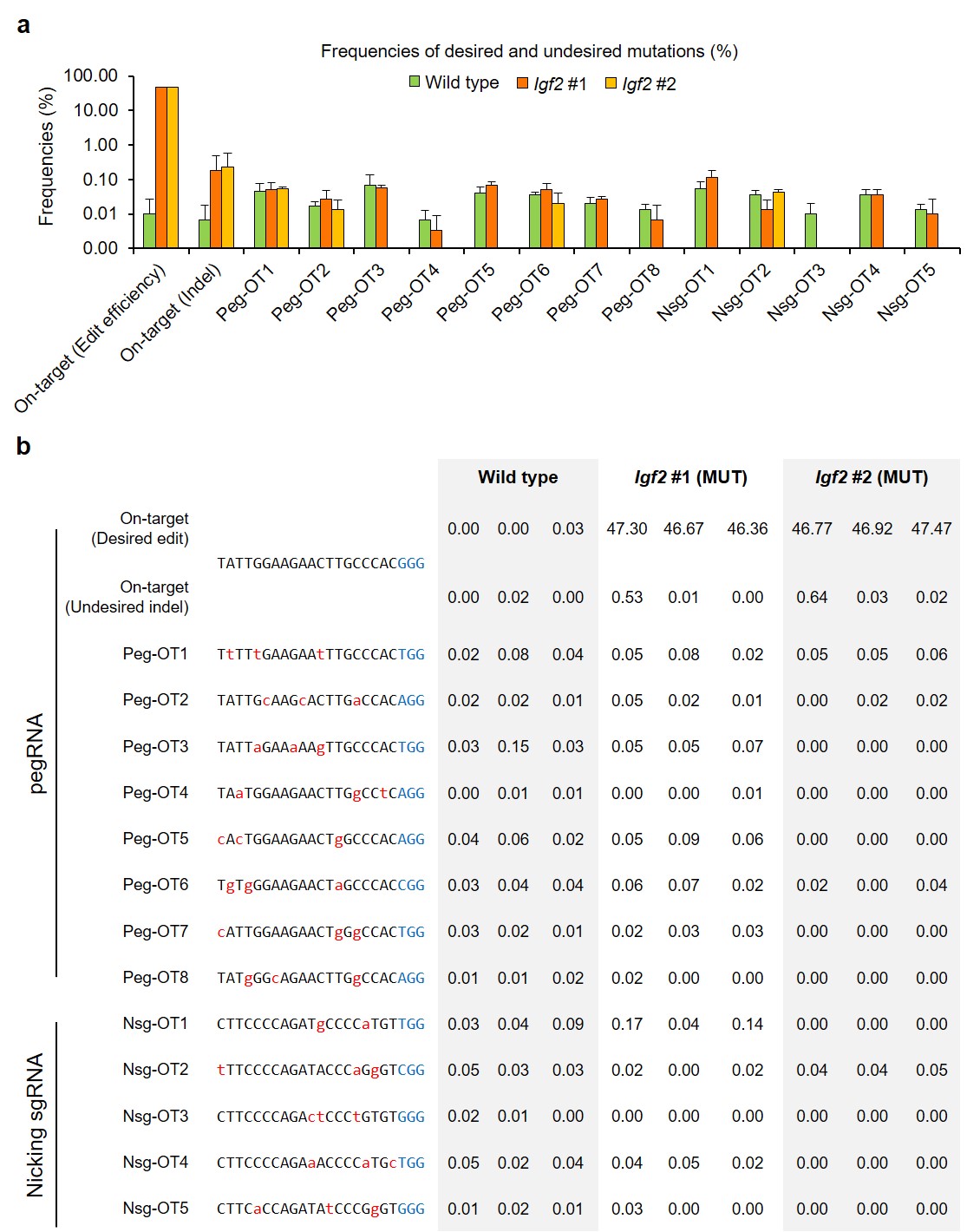
**

**Figure S7. Indel frequencies at the potential off-target sites of pegRNA and nsgRNA used for targeted mutagenesis of the *Igf2* target site.**

**Table S1. Targeted mutagenesis in mouse embryos.**

**
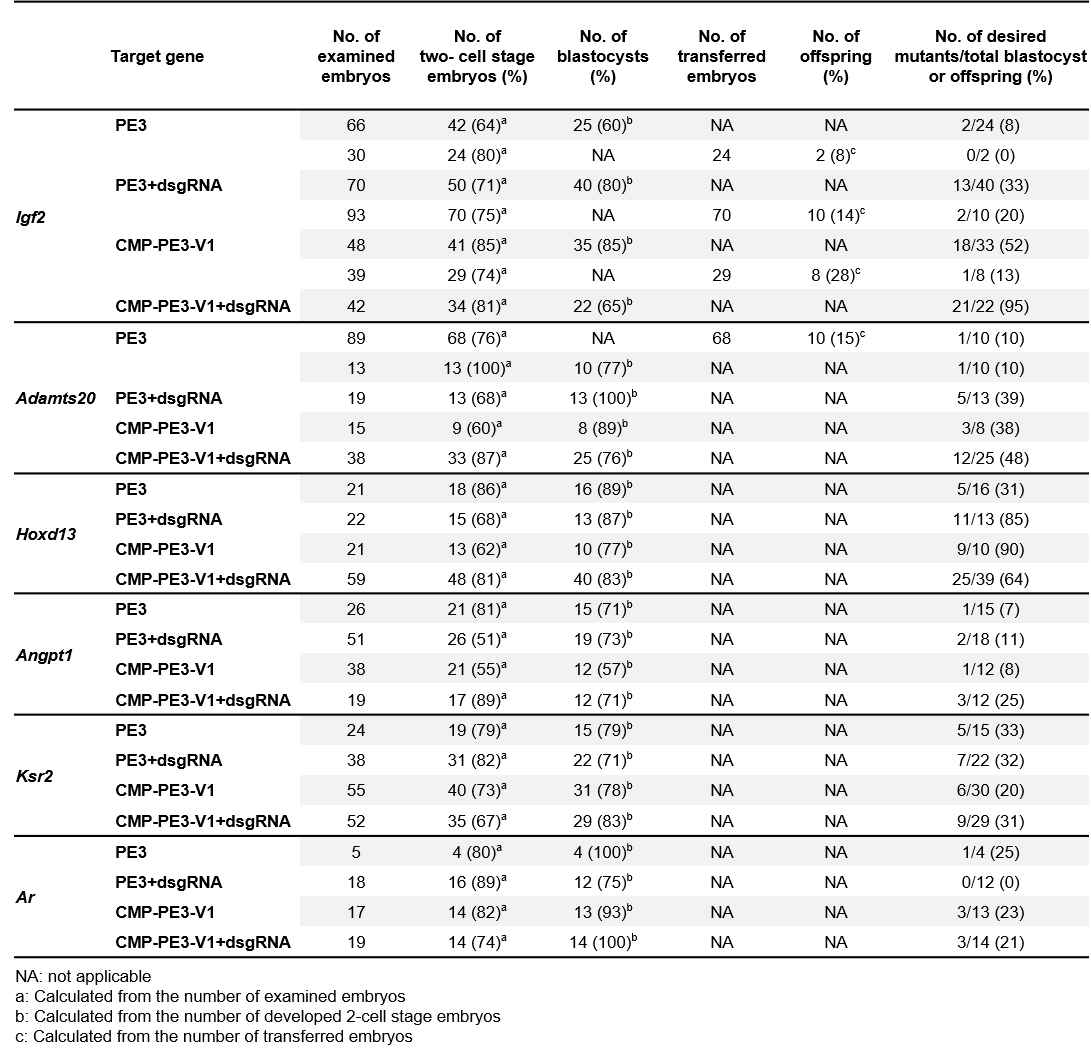
**

NA: not applicable

a: Calculated from the number of examined embryos

b: Calculated from the number of developed 2-cell stage embryos

c: Calculated from the number of transferred embryos

**Table S2. Sequences of pegRNAs, nsgRNAs, and dsgRNAs used in this study.**

| **pegRNA** | **Spacer (5' to 3')** | **Primer Binding Site**  **(5' to 3')** | **RT template (5' to 3')** | **PBS length (nt)** | **RT template length (nt)** |
| --- | --- | --- | --- | --- | --- |
| tdTomato_G to C / T ins_8-17 | CGCATGGAGGGCTCCATGAA | ATGGAGCC | GAACTCAGTGGCGGTTC | 8 | 17 |
| *Igf2*_G to C / TA ins_9-14 | TATTGGAAGAACTTGCCCAC | GGCAAGTTC | AGATACCGCGTGTA | 9 | 14 |
| *Adamts20*_CG to AA_11-13 | AGTGAATAAGAAGACGTACT | ACGTCTTCTTA | GACCGGCCTTAGT | 11 | 13 |
| *Casp1*_TAGG del_12-12 | GTCTTGTCTCTTATAGGAGA | CCTATAAGAGAC | ACCTCTTTCACT | 12 | 12 |
| *Hoxd13*_G to T_10-15 | GAGGCATACATCTCCATGGA | ATGGAGATGT | GACTGGTAGACCTCC | 10 | 15 |
| *Angpt1*_CGG to TGA_11-13 | CACATTGCCCATGTTGAATC | TCAACATGGGC | GATATAACTGAAT | 11 | 13 |
| *Ksr2*_TGAT ins_8-14 | TCCTGCCCTGGCTCCGTGGT | ACGGAGCC | GTGGCCCACCTGAT | 8 | 14 |
| *Ar*_G to T_13-13 | TCTCACTTGTGGCAGCTGCA | AGCTGCCACAAGT | AAGAAGACCTTGA | 13 | 13 |

| **Nicking sgRNA** | **sequence (5' to 3')** |
| --- | --- |
| tdTomato-nsgRNA | GCCCTCGATCTCGAACTCAG |
| *Igf2*-nsgRNA | CTTCCCCAGATACCGCGTGT |
| *Adamt20*-nsgRNA | AGCTCGTGTTCAAGGACATG |
| *Casp1*-nsgRNA | CTGTCAGAAGTCTTGTGCTC |
| *Hoxd13*-nsgRNA | GATCCTTGGCACAGTACACC |
| *Angpt1*-nsgRNA | GATATAACTGAATTCAACAT |
| *Ksr2*-nsgRNA | ACCTGATACGGAGCCAGGGC |
| *Ar*-nsgRNA | AGGGGAAAATATCAGGAAGT |

| **dsgRNA** | **Sequence (5' to 3')** |
| --- | --- |
| *Igf2*_dsgRNA_-34 | AGTCAGCAGGTTTC |
| *Igf2*_dsgRNA_-27 | AGGTGCTAGTCAGC |
| *Igf2*_dsgRNA_+7 | TGGAGACAGTCCGC |
| *Igf2*_dsgRNA_+22 | GGACGCCTGCGCAG |
| *Adamts20*_dsgRNA_-13 | TCAAGAGCACAGCC |
| *Adamts20*_dsgRNA_+14 | TTTCTGGTGCTTGG |
| *Adamts20*_dsgRNA_+24 | CGATTTTGACTTTC |
| *Adamts20*_dsgRNA_+41 | CTTCCGCTCACTTC |
| *Casp1*_dsgRNA_-13 | CTTTGACTTCTCTA |
| *Casp1*_dsgRNA_-12 | ACTTTGACTTCTCTA |
| *Casp1*_dsgRNA_+7 | CAGCAAATTCTTTC |
| *Casp1*_dsgRNA_+62 | GTATTCATGTCTCA |
| *Hoxd13*_dsgRNA_-12 | CCCGGATCCAAAAG |
| *Hoxd13*_dsgRNA_-11 | CACTTTTGGATCCG |
| *Hoxd13*_dsgRNA_+20 | GCTGTTCCACCCGT |
| *Hoxd13*_dsgRNA_+24 | CGGGTGGAACAGCC |
| *Angpt1*_dsgRNA_-15 | CGAAATCCAGAAAA |
| *Angpt1*_dsgRNA_-11 | ATCCAGAAAACGGA |
| *Angpt1*_dsgRNA_+14 | CTTCCAGAACACGA |
| *Angpt1*_dsgRNA_+17 | TTCCCGTCGTGTTC |
| *Ksr2*_dsgRNA_-12 | TCTCCAAACAAGAT |
| *Ksr2*_dsgRNA_+7 | TCCGGGGGGCACAC |
| *Ar*_dsgRNA_-15 | GGAGATGAAGCTTC |

**Table S3. Sequencies of the on-target and the potential off-target sites of pegRNA and nsgRNA used for targeted mutagenesis of the *Igf2* target site.** The off-target sites with up to 3 bp mismatches were selected using Cas-OFFinder.

|  | **Target** | **Gene** | **Sequence with PAM (5’ to 3’)** | **Chromosome** | **Position** | **Direction** |
| --- | --- | --- | --- | --- | --- | --- |
| pegRNA | On-target | *Igf2* | TATTGGAAGAACTTGCCCACGGG | chr7 | 142654011 | + |
|  | Peg-OT1 | Intergenic region | TtTTtGAAGAAtTTGCCCACTGG | chr7 | 103794877 | + |
|  | Peg-OT2 | Intergenic region | TATTGcAAGcACTTGaCCACAGG | chr4 | 92113105 | - |
|  | Peg-OT3 | Intergenic region | TATTaGAAaAAgTTGCCCACTGG | chr16 | 27823307 | - |
|  | Peg-OT4 | *Csdm3, Aim2* | TAaTGGAAGAACTTGgCCtCAGG | chr1 | 173360117 | + |
|  | Peg-OT5 | *Aopep* | cAcTGGAAGAACTgGCCCACAGG | chr13 | 63196984 | - |
|  | Peg-OT6 | Wdfy4 | TgTgGGAAGAACTaGCCCACCGG | chr14 | 32961677 | - |
|  | Peg-OT7 | Intergenic region | cATTGGAAGAACTgGgCCACTGG | chr9 | 26349102 | - |
|  | Peg-OT8 | *Ophn1* | TATgGGcAGAACTTGgCCACAGG | chrX | 98556772 | - |
| Nickng sgRNA | On-target | *Igf2* | CTTCCCCAGATACCCCGTGTAGG | chr7 | 142654028 | - |
|  | nsg-OT1 | *Dock3* | CTTCCCCAGATgCCCCaTGTTGG | chr9 | 106943872 | - |
|  | nsg-OT2 | *Olfr263* | tTTCCCCAGATACCCaGgGTCGG | chr13 | 21129729 | - |
|  | nsg-OT3 | *2610307P16Rik* | CTTCCCCAGActCCCtGTGTGGG | chr13 | 28531870 | - |
|  | nsg-OT4 | *9030622O22Rik* | CTTCCCCAGAaACCCCaTGcTGG | chr2 | 148005663 | + |
|  | nsg-OT5 | *Csmd3* | CTTCaCCAGATAtCCCGgGTGGG | chr15 | 47849475 | - |

**OT: Off-target**

**Table S4. Primer sequences used to amplify the target DNA in this study.**

|  | **1^st^ PCR** | | **2^nd^ PCR** | |
| --- | --- | --- | --- | --- |
| **Target** | **Forward (5’ to 3’)** | **Reverse (5’ to 3’)** | **Forward (5’ to 3’)** | **Reverse (5’ to 3’)** |
| tdTomato | CTTCCTCGTGCTTTACGGTATC | CGCGCATCTTCACCTTGTA | - | - |
| *Igf2* | GTGAGACAAAGAGACCACTCAC | CTCAAGAGGAGGTCACAGATTG | ACACTCTTTCCCTACACGACGCTCTTCCGATCTTAGATGGGAGCTCAGGCTAA | GTGACTGGAGTTCAGACGTGTGCTCTTCCGATCTGACGTTTGGCCTCTCTGAA |
| *Adamts20* | TACAGAGACACAAGCCATTCC | CCATCACAGACTCACCATTCA | ACACTCTTTCCCTACACGACGCTCTTCCGATCTCTACAGCTCGTGTTCAAGGA | GTGACTGGAGTTCAGACGTGTGCTCTTCCGATCTGAAATTCTTCCAGGCGGATTC |
| *Casp1* | CCTTGCTAGTTCTGTACTGGTG | ATCACCTTGGGCTTGTCTTT | ACACTCTTTCCCTACACGACGCTCTTCCGATCTTGTCCTTAGAGAAGTCAAAGTGTAA | GTGACTGGAGTTCAGACGTGTGCTCTTCCGATCTGGGCACTTCAAAGTGTTCATC |
| *Hoxd13* | CACTTCGGCAACGGTTACTA | CTGGTCAGGCCTGGAATATAAG | ACACTCTTTCCCTACACGACGCTCTTCCGATCTCTTCTACCAGGGCTACACAAG | GTGACTGGAGTTCAGACGTGTGCTCTTCCGATCTCTCCTACCTGGAAAGGATGATT |
| *Angpt1* | GAACAAAGCTAACAAATGGCTAGT | TGCTCCAGATGCTGAAGTTT | ACACTCTTTCCCTACACGACGCTCTTCCGATCTGCTGGCAGTACAATGACAGTT | GTGACTGGAGTTCAGACGTGTGCTCTTCCGATCTCGTGTGGAGCATCCCTTTG |
| *Ksr2* | GCAGACAAGGTGCAGAAGA | GGCACAAGTTCGGGTAGG | ACACTCTTTCCCTACACGACGCTCTTCCGATCTGGTGCTGATGGTGGATCTTT | GTGACTGGAGTTCAGACGTGTGCTCTTCCGATCTTCTGGGAGAGGTGGGTTC |
| *Ar* | GCTATTCCCTCAGAACCTGTTTA | GACCACCTACATCACCATCATT | ACACTCTTTCCCTACACGACGCTCTTCCGATCTCAGTACCAGGGACCATGTTT | GTGACTGGAGTTCAGACGTGTGCTCTTCCGATCTGTCACTGTCATTCTCTGCTAGG |

|  | **1^st^ PCR** | | | **2^nd^ PCR** | | |  |
| --- | --- | --- | --- | --- | --- | --- | --- |
| **Target** | **Forward (5’ to 3’)** | **Reverse (5’ to 3’)** | | **Forward (5’ to 3’)** | | **Reverse (5’ to 3’)** |  |
| Peg-OT1 | TTTGTCTTCCTGGGCATGAG | TTCAATGTGTCTAGCTATTAGGGAA | ACACTCTTTCCCTACACGACGCTCTTCCGATCTTTCCTGTGATTGGGCATCTC | | GTGACTGGAGTTCAGACGTGTGCTCTTCCGATCTAAGCCCTGTTCACTACTGATG | | |
| Peg-OT2 | CCAATGCTGTCCTCTGTAGAATG | GCGGTCTACCATTTCTTTAATGAGT | ACACTCTTTCCCTACACGACGCTCTTCCGATCTCAGTGGATGTTGGGAATCTCTA | | GTGACTGGAGTTCAGACGTGTGCTCTTCCGATCTGCAATTACAATAGCAGAAATGACAC | | |
| Peg-OT3 | GCTCCATCTCTCAACAGCATATAG | TACAGAGTCAACTCCAGCAGA | ACACTCTTTCCCTACACGACGCTCTTCCGATCTGGCTGTCAATGGGTTCTCTAC | | GTGACTGGAGTTCAGACGTGTGCTCTTCCGATCTCAGCTAGGCAAGCTACAAGAA | | |
| Peg-OT4 | CCAGGGCTTGTAAACCAGAA | CTCATTCTTTAAATATCAGGTGGCA | ACACTCTTTCCCTACACGACGCTCTTCCGATCTCACCATGGTCCCACTGTAAT | | GTGACTGGAGTTCAGACGTGTGCTCTTCCGATCTCCCTTAGAAATCCAGAGAAACTCTA | | |
| Peg-OT5 | CCAGGCTAGGCCACTATAA | AAGTCCTGGATTTGGTAATCTC | ACACTCTTTCCCTACACGACGCTCTTCCGATCTACACCACTACATCTCCTCAGAC | | GTGACTGGAGTTCAGACGTGTGCTCTTCCGATCTGGGAAGTCCTTCCTGGCT | | |
| Peg-OT6 | GGCAAGGAAGGAGATCTGTAAA | GAGATGGAGAGACAGCAAGTG | ACACTCTTTCCCTACACGACGCTCTTCCGATCTTTGTGGGCAGCTGTAATCTAA | | GTGACTGGAGTTCAGACGTGTGCTCTTCCGATCTGCAACAGGGAACACATAGGA | | |
| Peg-OT7 | GCCAGGGTCATTTGCAATAAAT | CAAGCAGCAATGGAAGGAAAG | ACACTCTTTCCCTACACGACGCTCTTCCGATCTCCTCCCATAGGCTCATTTGTT | | GTGACTGGAGTTCAGACGTGTGCTCTTCCGATCTAAGGCTGTTCGTATTGCATATTTAG | | |
| Peg-OT8 | GAGTCATTGACAGCGCACTA | CACCTTTCCCAGCTGAGTTT | ACACTCTTTCCCTACACGACGCTCTTCCGATCTTCTTGGAGCTGTAGCCTATCT | | GTGACTGGAGTTCAGACGTGTGCTCTTCCGATCTTGTCTTTCTCTGCCTTCCAATAG | | |
| nsg-OT1 | GAGATCCTGTCTTTGCTGGAAT | CTAAGAGTGTGTGCTTCCTTGT | ACACTCTTTCCCTACACGACGCTCTTCCGATCTCCTTCTCTGTCTGCATTCATTTG | | GTGACTGGAGTTCAGACGTGTGCTCTTCCGATCTCTCCTGGTACCCAATCCTAGA | | |
| nsg-OT2 | AGCTGAAGGACAGAGGATTAAAG | TGCCAAGACAACAGGATGAA | ACACTCTTTCCCTACACGACGCTCTTCCGATCTAGTGTGTTCATGTACATTTGGA | | GTGACTGGAGTTCAGACGTGTGCTCTTCCGATCTCTTCTCTTCCAGAGCCAACA | | |
| nsg-OT3 | AGAAGGAGCTATTACATACACTTAGAC | CTGTGTGGTAAATGACCTGGA | ACACTCTTTCCCTACACGACGCTCTTCCGATCTAAGATCGATGTTTGGGCTACA | | GTGACTGGAGTTCAGACGTGTGCTCTTCCGATCTGCTAAGTGGAAACTGCTCCT | | |
| nsg-OT4 | CTGCTCCAGACTAACAGGATTAC | CCCAGCACTTAGGAGGATAAAG | ACACTCTTTCCCTACACGACGCTCTTCCGATCTGGTTTAAGAGCCAAGTTCCTAGAAG | | GTGACTGGAGTTCAGACGTGTGCTCTTCCGATCTGGGAAGATAATGGACCAACATCAG | | |
| nsg-OT5 | AGTGTCAATACGGACTATGTTATCT | AGAATCTTCCAAATCAGGACTACT | ACACTCTTTCCCTACACGACGCTCTTCCGATCTTGCTCTGGCATTTGTATTAGG | | GTGACTGGAGTTCAGACGTGTGCTCTTCCGATCTACTAGTGGTATTAAGTACAGAGAGA | | |

**Table S5. Primer sequences used for real-time qPCR.**

| **Target** | **Forward (5’ to 3’)** | **Reverse (5’ to 3’)** |
| --- | --- | --- |
| Closed chromatin | GCAGCAGATGGCAAGTAATACTAAGAT | CCCTTATTCTCTGAGCATTAGACAGTTATA |
| Open chromatin | TGAGTCACAGCATCAGCATCGGGTCTCT | GGAGAGGGCCTTTTTCTCTTCAAGGTTC |
| *Igf2* | TCTCCAGGACGACTTCCCCAGA | CTCCAGGTGTCATATTGGAAGAAC |
| *Adamts20* | GTGGAATCAAGAGCACAGC | CAGTGAATAAGAAGACGTAC |
| *Casp1* | AAATCACTGGTCTTGTCTCTTATAG | CAGCAAATTCTTTCACCTCTTTC |
| *Hoxd13* | TCGGCACGAGGCATACATCTCCATG | CGTTGGCTAGCGTCCAGGACTG |
| *Angpt1* | CCAGAAAACGGAGGGAGAAGA | TAGGCACATTGCCCATGTTGA |
| *Ksr2* | TCTCTCCAAACAAGATTGGATCAT | TCTCCTGCCCTGGCTCC |

**Table S6. Amino acid sequences of CMP-PE-V1 and CMP-PE-V2.**

Purple: Bipartite NLS

Green: HN1

Yellow: Linker

Grey : nCas9

Light green: H1G

Blue: M-MLV RT

**CMP-PE-V1**

MKRTADGSEFESPKKKRKVMPKRKVSSAEGAAKEEPKRRSARLSAKPPAKVEAKPKKAAAKDKSSDKKVQTKGKRGAKGKQAEVANQETKEDLPAENGETKTEESPASDEAGEKEAKSDTGSGDKKYSIGLDIGTNSVGWAVITDEYKVPSKKFKVLGNTDRHSIKKNLIGALLFDSGETAEATRLKRTARRRYTRRKNRICYLQEIFSNEMAKVDDSFFHRLEESFLVEEDKKHERHPIFGNIVDEVAYHEKYPTIYHLRKKLVDSTDKADLRLIYLALAHMIKFRGHFLIEGDLNPDNSDVDKLFIQLVQTYNQLFEENPINASGVDAKAILSARLSKSRRLENLIAQLPGEKKNGLFGNLIALSLGLTPNFKSNFDLAEDAKLQLSKDTYDDDLDNLLAQIGDQYADLFLAAKNLSDAILLSDILRVNTEITKAPLSASMIKRYDEHHQDLTLLKALVRQQLPEKYKEIFFDQSKNGYAGYIDGGASQEEFYKFIKPILEKMDGTEELLVKLNREDLLRKQRTFDNGSIPHQIHLGELHAILRRQEDFYPFLKDNREKIEKILTFRIPYYVGPLARGNSRFAWMTRKSEETITPWNFEEVVDKGASAQSFIERMTNFDKNLPNEKVLPKHSLLYEYFTVYNELTKVKYVTEGMRKPAFLSGEQKKAIVDLLFKTNRKVTVKQLKEDYFKKIECFDSVEISGVEDRFNASLGTYHDLLKIIKDKDFLDNEENEDILEDIVLTLTLFEDREMIEERLKTYAHLFDDKVMKQLKRRRYTGWGRLSRKLINGIRDKQSGKTILDFLKSDGFANRNFMQLIHDDSLTFKEDIQKAQVSGQGDSLHEHIANLAGSPAIKKGILQTVKVVDELVKVMGRHKPENIVIEMARENQTTQKGQKNSRERMKRIEEGIKELGSQILKEHPVENTQLQNEKLYLYYLQNGRDMYVDQELDINRLSDYDVDAIVPQSFLKDDSIDNKVLTRSDKNRGKSDNVPSEEVVKKMKNYWRQLLNAKLITQRKFDNLTKAERGGLSELDKAGFIKRQLVETRQITKHVAQILDSRMNTKYDENDKLIREVKVITLKSKLVSDFRKDFQFYKVREINNYHHAHDAYLNAVVGTALIKKYPKLESEFVYGDYKVYDVRKMIAKSEQEIGKATAKYFFYSNIMNFFKTEITLANGEIRKRPLIETNGETGEIVWDKGRDFATVRKVLSMPQVNIVKKTEVQTGGFSKESILPKRNSDKLIARKKDWDPKKYGGFDSPTVAYSVLVVAKVEKGKSKKLKSVKELLGITIMERSSFEKNPIDFLEAKGYKEVKKDLIIKLPKYSLFELENGRKRMLASAGELQKGNELALPSKYVNFLYLASHYEKLKGSPEDNEQKQLFVEQHKHYLDEIIEQISEFSKRVILADANLDKVLSAYNKHRDKPIREQAENIIHLFTLTNLGAPAAFKYFDTTIDRKRYTSTKEVLDATLIHQSITGLYETRIDLSQLGGDLEGGGGSSTDHPKYSDMIVAAIQAEKNRAGSSRQSIQKYIKSHYKVGENADSQIKLSIKRLVTTGVLKQTKGVGASGSFRLAKSDEPSGGSSGGSSGSETPGTSESATPESSGGSSGGSSTLNIEDEYRLHETSKEPDVSLGSTWLSDFPQAWAETGGMGLAVRQAPLIIPLKATSTPVSIKQYPMSQEARLGIKPHIQRLLDQGILVPCQSPWNTPLLPVKKPGTNDYRPVQDLREVNKRVEDIHPTVPNPYNLLSGLPPSHQWYTVLDLKDAFFCLRLHPTSQPLFAFEWRDPEMGISGQLTWTRLPQGFKNSPTLFNEALHRDLADFRIQHPDLILLQYVDDLLLAATSELDCQQGTRALLQTLGNLGYRASAKKAQICQKQVKYLGYLLKEGQRWLTEARKETVMGQPTPKTPRQLREFLGKAGFCRLFIPGFAEMAAPLYPLTKPGTLFNWGPDQQKAYQEIKQALLTAPALGLPDLTKPFELFVDEKQGYAKGVLTQKLGPWRRPVAYLSKKLDPVAAGWPPCLRMVAAIAVLTKDAGKLTMGQPLVILAPHAVEALVKQPPDRWLSNARMTHYQALLLDTDRVQFGPVVALNPATLLPLPEEGLQHNCLDILAEAHGTRPDLTDQPLPDADHTWYTDGSSLLQEGQRKAGAAVTTETEVIWAKALPAGTSAQRAELIALTQALKMAEGKKLNVYTDSRYAFATAHIHGEIYRRRGWLTSEGKEIKNKDEILALLKALFLPKRLSIIHCPGHQKGHSAEARGNRMADQAARKAAITETPDTSTLLIENSSPSGGSKRTADGSEFEPKKKRKV

**CMP-PE-V2**

MKRTADGSEFESPKKKRKVMPKRKVSSAEGAAKEEPKRRSARLSAKPPAKVEAKPKKAAAKDKSSDKKVQTKGKRGAKGKQAEVANQETKEDLPAENGETKTEESPASDEAGEKEAKSDTGSGDKKYSIGLDIGTNSVGWAVITDEYKVPSKKFKVLGNTDRHSIKKNLIGALLFDSGETAEATRLKRTARRRYTRRKNRICYLQEIFSNEMAKVDDSFFHRLEESFLVEEDKKHERHPIFGNIVDEVAYHEKYPTIYHLRKKLVDSTDKADLRLIYLALAHMIKFRGHFLIEGDLNPDNSDVDKLFIQLVQTYNQLFEENPINASGVDAKAILSARLSKSRRLENLIAQLPGEKKNGLFGNLIALSLGLTPNFKSNFDLAEDAKLQLSKDTYDDDLDNLLAQIGDQYADLFLAAKNLSDAILLSDILRVNTEITKAPLSASMIKRYDEHHQDLTLLKALVRQQLPEKYKEIFFDQSKNGYAGYIDGGASQEEFYKFIKPILEKMDGTEELLVKLNREDLLRKQRTFDNGSIPHQIHLGELHAILRRQEDFYPFLKDNREKIEKILTFRIPYYVGPLARGNSRFAWMTRKSEETITPWNFEEVVDKGASAQSFIERMTNFDKNLPNEKVLPKHSLLYEYFTVYNELTKVKYVTEGMRKPAFLSGEQKKAIVDLLFKTNRKVTVKQLKEDYFKKIECFDSVEISGVEDRFNASLGTYHDLLKIIKDKDFLDNEENEDILEDIVLTLTLFEDREMIEERLKTYAHLFDDKVMKQLKRRRYTGWGRLSRKLINGIRDKQSGKTILDFLKSDGFANRNFMQLIHDDSLTFKEDIQKAQVSGQGDSLHEHIANLAGSPAIKKGILQTVKVVDELVKVMGRHKPENIVIEMARENQTTQKGQKNSRERMKRIEEGIKELGSQILKEHPVENTQLQNEKLYLYYLQNGRDMYVDQELDINRLSDYDVDAIVPQSFLKDDSIDNKVLTRSDKNRGKSDNVPSEEVVKKMKNYWRQLLNAKLITQRKFDNLTKAERGGLSELDKAGFIKRQLVETRQITKHVAQILDSRMNTKYDENDKLIREVKVITLKSKLVSDFRKDFQFYKVREINNYHHAHDAYLNAVVGTALIKKYPKLESEFVYGDYKVYDVRKMIAKSEQEIGKATAKYFFYSNIMNFFKTEITLANGEIRKRPLIETNGETGEIVWDKGRDFATVRKVLSMPQVNIVKKTEVQTGGFSKESILPKRNSDKLIARKKDWDPKKYGGFDSPTVAYSVLVVAKVEKGKSKKLKSVKELLGITIMERSSFEKNPIDFLEAKGYKEVKKDLIIKLPKYSLFELENGRKRMLASAGELQKGNELALPSKYVNFLYLASHYEKLKGSPEDNEQKQLFVEQHKHYLDEIIEQISEFSKRVILADANLDKVLSAYNKHRDKPIREQAENIIHLFTLTNLGAPAAFKYFDTTIDRKRYTSTKEVLDATLIHQSITGLYETRIDLSQLGGDSGGSSGGSSGSETPGTSESATPESSGGSSGGSSTLNIEDEYRLHETSKEPDVSLGSTWLSDFPQAWAETGGMGLAVRQAPLIIPLKATSTPVSIKQYPMSQEARLGIKPHIQRLLDQGILVPCQSPWNTPLLPVKKPGTNDYRPVQDLREVNKRVEDIHPTVPNPYNLLSGLPPSHQWYTVLDLKDAFFCLRLHPTSQPLFAFEWRDPEMGISGQLTWTRLPQGFKNSPTLFNEALHRDLADFRIQHPDLILLQYVDDLLLAATSELDCQQGTRALLQTLGNLGYRASAKKAQICQKQVKYLGYLLKEGQRWLTEARKETVMGQPTPKTPRQLREFLGKAGFCRLFIPGFAEMAAPLYPLTKPGTLFNWGPDQQKAYQEIKQALLTAPALGLPDLTKPFELFVDEKQGYAKGVLTQKLGPWRRPVAYLSKKLDPVAAGWPPCLRMVAAIAVLTKDAGKLTMGQPLVILAPHAVEALVKQPPDRWLSNARMTHYQALLLDTDRVQFGPVVALNPATLLPLPEEGLQHNCLDILAEAHGTRPDLTDQPLPDADHTWYTDGSSLLQEGQRKAGAAVTTETEVIWAKALPAGTSAQRAELIALTQALKMAEGKKLNVYTDSRYAFATAHIHGEIYRRRGWLTSEGKEIKNKDEILALLKALFLPKRLSIIHCPGHQKGHSAEARGNRMADQAARKAAITETPDTSTLLIENSSPSGGSLEGGGGSSTDHPKYSDMIVAAIQAEKNRAGSSRQSIQKYIKSHYKVGENADSQIKLSIKRLVTTGVLKQTKGVGASGSFRLAKSDEPKRTADGSEFEPKKKRKV
