# Supplementary material for: Targeted mutagenesis in mouse cells and embryos using an enhanced prime editor
Source: Genome Biol. 2021 Jun 3;22:170. doi: 10.1186/s13059-021-02389-w (PMC8173820; doi:10.1186/s13059-021-02389-w)
Supplement: Supplementary file 2 — Additional file 2. Review history. [file 13059_2021_2389_MOESM2_ESM.docx]

Review History

**First round of review**

**Reviewer 1**

**Are you able to assess all statistics in the manuscript, including the appropriateness of statistical tests used?** Yes, and I have assessed the statistics in my report.

**Comments to author:**

This study by Park et al. demonstrated the feasibility of increasing prime editing efficiency by chromatin accessibility approach. Overall, this study is useful for enhancing the prime editing, while further evidences are needed to strengthen the study.
Major concerns:
1.     Please reorganized the manuscript. For example, optimizing PE with cell lines, then testing the optimized PE in embryos, and in mice.
2.     Only two sites are tested. For improvement, at least six sites are needed for the comprehensive characterization.
3.     The author should provide direct evidences to establish the link between chromatin accessibility and prime editing efficiency, such as ChIP-seq, Dnase-seq.

Minor concerns:
1.     The information of repeat should include in the statistics analysis in the methods.
2.     The dwarf phenotype induced by lgf2 mutation in this study is discussed limited, usually body length and body weight should be presented to enrich the results.
3.     Different groups in Fig. 2a should be annotated in figure legends in detail.

**Reviewer 2**

**Are you able to assess all statistics in the manuscript, including the appropriateness of statistical tests used?** Yes, and I have assessed the statistics in my report.

**Comments to author:**

Brief Summary:
In this manuscript, the authors provided technical solutions to low efficiency of prime editing system by additionally introducing CMP and dsgRNA modules, separately or as a fusion peptide to PE. They provided evidence for the improvement of PE efficiency through results obtained from cell line and embryo experiments. The authors stated that CMP was highly effective in modulating PE activity and that the effect of dsgRNA is cell-type and locus-dependent. In addition, the CMP and dsgRNA may evoke a synergic effect on the generation of model animals and cell research.

Overall impression of the work
The degree of improvements of PE efficiency by CMP and/or dsgRNA was not marginal and, for some cases, it increased the PE efficiency by more than 10-fold. The impact of this technology is not small. Nonetheless, more evidence needs to be complemented for researchers or scientists to adopt this technology with high assurance. There are some major and minor points regarding those issues as follows:

Specific Comments
Comment 1:
More detailed description on dsgRNA is required for clear understanding of how dsgRNA works to improve PE activity.

Comment 2:
In Fig. S6, I can't be convinced whether dsgRNA is effective in improving PE activity. dsgRNA was effective for a certain gene, but not other in the same cell line. And, the effects are different depending on the cell lines. Furthermore, dsgRNA appears to even nullify the CMP effect, to some degree (Fig. 2d). Could you provide more robust data that support your claim that dsgRNA is effective? As you stated, the effects may be cell type-dependent. But there should be an artifact that behaves as a confounding factor. Use of a scrambled dsgRNA could be one of the tactics to address the issue.

Comment 3:
The incorporation of CMP into PE modules may change the overall structure of nCas9-RT fusion proteins. Thus, we may imagine that the CMP effects would be derived from such switch of the PE structure, possibly to a more favorable one. To rule out the possibility, the null variants of HN1 or H1G, if any, could be used as a negative control.

Comment 4:
The CMP effects are thought to be due to the modulatory effect of chromatin architecture. Could you take any validation action to address the expected cause for the effects? Plus, the CMP is effective to both euchromatin and heterochromatin structures? Or any one of them?

Comment 5:
In Figs. S2 and S3, the authors claimed that PE3 showed a poor PE activity in both NIH3T3 cell line and embryos. However, more factors possibly affect the final PE activity including transfection efficiency for cell line, Cas9 cleavage activity and, finally PE activity. The authors should show indel frequency of Cas9 at the Adamts20 and Igf2 loci to exclude the possibility of low efficiency in transfection or dsDNA breaks.

Comment 6:
A sample size analysis would be required to assess the effect of CMP or dsgRNA. Or, an increased sample size, at least, would be necessary to validate the effects of those modulators.

Comment 7:
PE activity is significantly varied depending on the length of PBS and RT. That means PE activity can be increased by simple modulation of those length. Could you compare the effects of CMP and/or dsgRNA with those of the PBS/RT modulations? Furthermore, the CMP/dsgRNA are still effective for the PBS/RT optimized PE activity? If it's true, the CMP/dsgRNA effects are more meaningful.

Minor points:

I cannot find any difference in the Sanger sequencing result in Fig.1e.

I cannot find the Fig. S9 data in the supplementary material.

In line 41, the authors stated that it still cannot make eight transversion mutations such as C to A, C to 42 G, G to C, G to T, A to C, A to T, T to A, and T to G. However, recent studies shows a technical adavances to overcome such hurdles, such as Kurt et al, Nat. Biotech (2021). The sentence should be revised considering the recent technical improvements.

In line 44: expanded -> extended

In line 57 and thereafter, the unit of length should be noted, such as length of 8 nt.

In line 58: the PAM sequence of pegRNA was changed… -> pegRNA was designed to remove the PAM sequence on the non-target strand…

**Authors Response**

**Point-by-point responses to the reviewers’ comments:**

**We would like to thank you for helpful comments. Following the editor's suggestions, we have now added new data in the content below to the revised manuscript. 1) To validate the advanced prime editor's efficiency in NIH3T3 cells, C2C12 cells, and mouse embryos, we added data by increasing the target site by up to six. 2) We also increased the sample size from n=3 to n=5 at all targets. 3) To show the effects of CMP or proximal dsgRNA on chromatin architecture, we tested DNase I digestion assay and qPCR. We show here direct evidence that the use of CMP-PE or dsgRNA can improve editing efficiency by unraveling chromatin structures and improving chromatin accessibility. The results have been added to Fig. 2g, and the manuscript has been revised. 4) We added bodyweight data of *Igf2* mutant mice two weeks after birth to Fig. 1e. We also added PE optimization tests in the mouse cell line at the supplementary data Figure S2. We also have modified the main text accordingly. Please find our point-by-point responses to the review’s criticisms below.**

**Reviewer #1:**

This study by Park et al. demonstrated the feasibility of increasing prime editing efficiency by chromatin accessibility approach. Overall, this study is useful for enhancing the prime editing, while further evidences are needed to strengthen the study.

**Major concerns:**
1. Please reorganized the manuscript. For example, optimizing PE with cell lines, then testing the optimized PE in embryos, and in mice.

**Response: We agree with your opinion. However, the production of a mouse model was a study to determine whether it could be applied to mice using PE and PE+dsgRNA. We have rearranged the manuscript according to your comments, excluding only mouse generation data using PE and PE + dsgRNA. We also added optimization data for PBS and RT length at the cell level before embryo testing in Supplementary Fig. S2 to fit the flow.**

2. Only two sites are tested. For improvement, at least six sites are needed for the comprehensive characterization.

**Response: We added data using additional target sites in mouse cells (*Angpt1* and *Ksr2* target sites) and embryos (*Adamts20, Hoxd13, Angpt1, Ksr2*, and *Ar* target sites). Data can be found in Supplementary Fig. S5, S6, S7, S8, and Supplementary Fig. Table S1.**

3. The author should provide direct evidences to establish the link between chromatin accessibility and prime editing efficiency, such as ChIP-seq, Dnase-seq.
**Response: We performed DNase I digestion assay and qPCR to establish the association between chromatin accessibility and prime editing efficiency. DNase I digestion analysis and qPCR are methods used in many studies to confirm chromatin structure states (Ling G et al. Methods Mol Biol. 2013, and Kim D, et al. Genome Res. 2018).**

**To determine whether CMP-PE and dsgRNA can improve chromatin accessibility by unraveling the chromatin structure of the target site, we performed DNaseI digestion assay and qPCR to verify the chromatin status of each target site. In the NIH/3T3 cell line, target sites of *Igf2*, *Adamts20*, and *Hoxd13* have appeared as closed chromatin relatively, and in C2C12, all targets except *Igf2* were open chromatin. This result suggests that even if the target sequence is the same for the two cell lines, the chromatin structure states are different. Also, we analyzed whether CMP-PE or dsgRNA could alter the chromatin status at the *Igf2* target site, a representative target of closed chromatin structure. As a result, we identified that the closed chromatin structure was gradually changed to open chromatin by CMP-PE, dsgRNA, or CMP-PE+dsgRNA compared to PE. These results are direct evidence that the use of CMP-PE or dsgRNA can improve editing efficiency by unraveling chromatin structures and improving chromatin accessibility. Also, we added the content to lines 151 to 161 of the manuscript on p. 7. We have added this data to Fig. 2f, 2g, Supplementary Fig. S9.**

**Minor concerns:**
1. The information of repeat should include in the statistics analysis in the methods.

**Response: We have moved recurring information to statistical analysis in the method section.**

2. The dwarf phenotype induced by lgf2 mutation in this study is discussed limited, usually body length and body weight should be presented to enrich the results.

**Response: We agree with your opinion. We added bodyweight data of *Igf2* mutant mice two weeks after birth to Fig. 1g. Also, we changed Fig. 1f to a picture of an *Igf2* mutant mouse with a scale bar to identify body size.**

3. Different groups in Fig. 2a should be annotated in figure legends in detail.
**Response: We have added details of the legend in Fig. 2a. We have rephrased the sentence as follows: “(a) Diagrams of Prime editor (PE), Chromatin-modulating peptide-Prime editor-Variant1 (CMP-PE-V1), and Chromatin-modulating peptide-Prime editor-Variant2 (CMP-PE-V2) construct. PE consists of nCas9 (H840A) and M-MLV RT. CMP-PE-V1 has HN1 at the N-terminus of nCas9 and H1G at the C-terminus of nCas9 in the PE structure. CMP-PE-V2 has HN1 at the N-terminus of nCas9 and H1G at the C-terminus of M-MLV RT in the PE structure. HN1, High-mobility group nucleosome binding domain 1; H1G, Histone H1 central globular domain.”**

**Reviewer #2: Brief Summary:**
In this manuscript, the authors provided technical solutions to low efficiency of prime editing system by additionally introducing CMP and dsgRNA modules, separately or as a fusion peptide to PE. They provided evidence for the improvement of PE efficiency through results obtained from cell line and embryo experiments. The authors stated that CMP was highly effective in modulating PE activity and that the effect of dsgRNA is cell-type and locus-dependent. In addition, the CMP and dsgRNA may evoke a synergic effect on the generation of model animals and cell research.

**Overall impression of the work**
The degree of improvements of PE efficiency by CMP and/or dsgRNA was not marginal and, for some cases, it increased the PE efficiency by more than 10-fold. The impact of this technology is not small. Nonetheless, more evidence needs to be complemented for researchers or scientists to adopt this technology with high assurance. There are some major and minor points regarding those issues as follows:

**Specific Comments**
Comment 1:
More detailed description on dsgRNA is required for clear understanding of how dsgRNA works to improve PE activity.
**Response: We agree with your opinion. On p. 4, lines 86-92, we have now commented that “In one attempt to improve the editing efficiency of PE, based on the idea of proxy-CRISPR, we employed dsgRNAs instead of catalytically dead endonuclease to unwind the chromatin structure of target sites. dsgRNA is 14 or 15 nt guide RNA that shows inactivated catalysis yet binds to the target site guiding Cas endonuclease. Thus we hypothesized that PE may play two roles. One is that prime editing at the target site with pegRNAs, and the other is modulating chromatin neighboring the target site with dsgRNAs. We designed proximal dsgRNAs adjacent to Igf2 and Adamts20 target sites in the range of 7–62 nucleotide positions from the spacer of pegRNA.”**

Comment 2:
In Fig. S6, I can't be convinced whether dsgRNA is effective in improving PE activity. dsgRNA was effective for a certain gene, but not other in the same cell line. And, the effects are different depending on the cell lines. Furthermore, dsgRNA appears to even nullify the CMP effect, to some degree (Fig. 2d). Could you provide more robust data that support your claim that dsgRNA is effective? As you stated, the effects may be cell type-dependent. But there should be an artifact that behaves as a confounding factor. Use of a scrambled dsgRNA could be one of the tactics to address the issue.

**Response: We have shown that the editing efficiency is different for each proximal dsgRNA site. And a screening process for proximal dsgRNA is required in each target and cell type to induce efficient targeted mutagenesis (Supplementary Fig. S5). Also, we have shown in DNase I digestion assay and qPCR data that for each cell line, even if the target sequence is the same, the state of the chromatin structure can be different (Supplementary Fig. S9). The prime editor using proximal dsgRNA can improve editing efficiency by opening the chromatin structure of the target site. However, simultaneous treatment of CMP-PE3 and proximal dsgRNA is more effective on targets with a closed chromatin structure (Fig. 2b, c, and Supplementary Fig. S7). We added data in the supplementary Fig. S5, S7, and S9.**

**We also used scrambled dsgRNA (sdsgRNA) to identify editing efficiency. PE3+sdsgRNA showed no difference in editing efficiency compared to PE3. We added data in Supplementary Fig. S5.**

Comment 3:
The incorporation of CMP into PE modules may change the overall structure of nCas9-RT fusion proteins. Thus, we may imagine that the CMP effects would be derived from such switch of the PE structure, possibly to a more favorable one. To rule out the possibility, the null variants of HN1 or H1G, if any, could be used as a negative control.

**Response: Ding X. et al. (2019 CRISPR J.) showed the effect of the null variants of HN1 or H1G at the N-terminus or C-terminus of S. pyogenes Cas9 (SpyCas9) in the Supplementary Table S1 of the paper. Interestingly, variants of human HMGN1 (HN1) or Histone H1 central globular domain (H1G) contributed to increasing SpyCas9 activity by a score of up to 2.3-fold change compared to the null variant in the K562 cell line. Overall, we can expect similar results because the Prime editor uses the SpyCas9 nickase variant.**

Comment 4:
The CMP effects are thought to be due to the modulatory effect of chromatin architecture. Could you take any validation action to address the expected cause for the effects? Plus, the CMP is effective to both euchromatin and heterochromatin structures? Or any one of them?
**Response: We tested DNase I digestion assays and qPCR to confirm the association between chromatin structure effects on CMP editing efficiency. DNase I digestion analysis and qPCR are methods used in many studies to confirm chromatin structure states (Ling G et al. Methods Mol Biol. 2013, and Kim D, et al. Genome Res. 2018).**

**We analyzed whether CMP could alter the chromatin state of the *Igf2,* a closed chromatin target. As a result, we identified that the closed chromatin structure was gradually changed to open chromatin by CMP-PE compared to PE. These results are direct evidence that the use of CMP-PE can improve editing efficiency by unraveling chromatin structures and improving chromatin accessibility. And the CMP is effective in both euchromatins (open chromatin) targets and heterochromatin (closed chromatin) targets. We added data in Fig. 2g, supplementary data Fig. S6, and S9.**

Comment 5:
In Figs. S2 and S3, the authors claimed that PE3 showed a poor PE activity in both NIH3T3 cell line and embryos. However, more factors possibly affect the final PE activity including transfection efficiency for cell line, Cas9 cleavage activity and, finally PE activity. The authors should show indel frequency of Cas9 at the Adamts20 and Igf2 loci to exclude the possibility of low efficiency in transfection or dsDNA breaks.

**Response: We agree with your opinion. In NIH3T3 and C2C12 cell lines, transfection efficiency was analyzed using the tdTomato expression vector via electroporation in the same manner as in the prime editor. This figure shows the efficiency of electroporation in NIH3T3 or C2C12 cell lines after 24 hours. We identified that electroporation efficiency was no problem through the expression of tdTomato in most of the cells.**

**Next, we tested editing efficiency using SpCas9 expression vector with each *Igf2* and *Adamts20* target nicking sgRNA (nsgRNA) to identify Cas9 and each target cleavage activity. Both the indel frequencies using Cas9 at the *Adamts20* and *Igf2* loci showed high efficiency up to 59.55 % and 88.44 %, which can be seen as a result that can exclude the possibility of low efficiency in the transfection or dsDNA breaks. On the other hand, SpCas9 with dead sgRNA (dsgRNA) did not generate indels.**

Comment 6:
A sample size analysis would be required to assess the effect of CMP or dsgRNA. Or, an increased sample size, at least, would be necessary to validate the effects of those modulators.

**Response: Sample size analysis was performed using previous experimental data using SPSS. The data of nine groups for the Adamta20 target sites was used for sample size analysis. The results of ANOVA showed that the effect size f was 0.976, the total sample size was 27, and the actual power was 0.863. This means that our experiment requires at least three independent experiments for each group. In addition, we increased the sample size from n = 3 to n = 5 at all target sites.**

Comment 7:
PE activity is significantly varied depending on the length of PBS and RT. That means PE activity can be increased by simple modulation of those length. Could you compare the effects of CMP and/or dsgRNA with those of the PBS/RT modulations? Furthermore, the CMP/dsgRNA are still effective for the PBS/RT optimized PE activity? If it's true, the CMP/dsgRNA effects are more meaningful.

**Response: We performed PE optimization experiments using PE3 or PE3 + dsgRNA in NIH/3T3 cell lines. PBS9-RT14 and PBS11-RT13 were selected as optimal pegRNAs for Igf2 and Adamts20 targets, respectively. We added data at the supplementary data Fig. S2. We have now commented on p. 4-5, lines 78-96.**

**Figure S2. Optimization of editing efficiency using various lengths of pegRNAs and proximal dsgRNA at the *Igf2* and *Adamts20* target sites.** (a, b) Design of mutagenesis on the Igf2 (a) and Adamts20 (b) target genes. (c, d) Comparison of editing efficiencies between PE3 and CMR-PE3 with diverse combinations of PBS lengths and RT template lengths in NIH/3T3 cells. Undesired indel frequencies are presented in parentheses. Data and error bars show mean±s.d. of three independent biological replicates.

**Minor points:**
I cannot find any difference in the Sanger sequencing result in Fig.1e.

**Response: This data indicates a potential positive off-target site in whole-genome sequencing. Here, we performed Sanger sequencing using the genomic DNA of a mutant *Igf2* mouse to identify an actual off-target site, and through the results, we confirmed that it was a false positive. Therefore, it is correct that there is no difference in the Sanger sequencing data in Figure 1e.**

I cannot find the Fig. S9 data in the supplementary material.

**Response: We have now revised the manuscript and Figure.**

In line 41, the authors stated that it still cannot make eight transversion mutations such as C to A, C to 42 G, G to C, G to T, A to C, A to T, T to A, and T to G. However, recent studies show a technical adavances to overcome such hurdles, such as Kurt et al., Nat. Biotech (2021). The sentence should be revised considering the recent technical improvements.

**Response: The reviewer's opinion is correct. We have now modified the phrase on p.2, lines 40-41 as follows: “A recent study has also reported a C-to-G base editor (CGBE1) that enables C-to-G base transversion in human cells.” We also added this paper (Kurt et al., Nat. Biotech (2021) to reference 6.**

In line 44: expanded -> extended

**Response: We have now corrected “by an extended form of prime editing guide RNA (pegRNA)” to “by a prime editing guide RNA (pegRNA).”**

In line 57 and thereafter, the unit of length should be noted, such as length of 8 nt.

**Response: We have added the unit of length (nt) in line 59.**

In line 58: the PAM sequence of pegRNA was changed… -> pegRNA was designed to remove the PAM sequence on the non-target strand…

**Response: We have revised the sentence from ‘the PAM sequence of pegRNA was changed’ to ‘pegRNA was designed to remove the PAM sequence on the non-target strand…’ in line 60.**

**Second round of review**

**Reviewer 1**

The revised manuscript by Park et al. showed well preparation for revision, and the authors also responded with corresponding new data. However, the manuscript still needs well-organization before being finally accepted.

Comments/Suggestions:
1.     I insist at least six sites are needed for the comprehensive characterization in Fig. 2.
2.     The manuscript should be prepared carefully. For example, the logic of a genome editing story should be cell line, mouse embryo, mouse offspring, off target analysis.
3.     Some necessary information, including the summary of embryos used during microinjection, should be provided.
4.     Professional language editing is required.

**Reviewer 2**

The revised manuscript is evaluated to provide reasonable rebuttals to the previous claims, except for Comment 3.
The effect of CMP on PE activity may be interpreted from the two aspects: One is from the increased nickase activity, and the other from improved downstream process including RT and ligation. I meant by the previous claim that the incorporated CMP may have a positive effect on RT via changes in overall structure of the PE module. However, the authors considered the CMP effects only with respect to increased nickase activity. Yes, CMP possibly improves nickase activity. But, could you rule out the possibility that CMP may favore the RT process via the structural fitness effects? This address can only be made with the incorporation of a null mutant of CMP to nCas9 and nCas9-RTase (PE) and comparison of the results.

**Authors Response**

**Point-by-point responses to the reviewers’ comments:**

**We would like to thank you for your helpful comments. Following the editor's suggestions, we have provided a reference for our dataset in the SRA and cited it in the Availability of Data and Materials section. We also confirmed authorship from all co-authors and uploaded a file related to the Change in Authorship form. We have removed the line numbers from the manuscript and uploaded the main figures separately. We have also provided subsections containing all additional file names and titles.**

**Following the reviewer's suggestions, we have revised and rearranged the abstract, main manuscript, and figures accordingly. In addition, our manuscript was revised by a professional native English editor. Please find our point-by-point responses to the reviewer’s criticisms below.**

**Reviewer reports:**

**Reviewer #1:**

The revised manuscript by Park et al. showed well preparation for revision, and the authors also responded with corresponding new data. However, the manuscript still needs well-organization before being finally accepted.

Comments/Suggestions:

1. I insist at least six sites are needed for the comprehensive characterization in Fig. 2.

**Response: We agree with your comments. We have moved the data for six additional target sites performed on mouse cells and embryos to Fig. 2.**

2. The manuscript should be prepared carefully. For example, the logic of a genome editing story should be cell line, mouse embryo, mouse offspring, off target analysis.

**Response: We have rearranged the abstract, manuscript, and figures according to your feedback. We believe that our revised manuscript and figures will address your concerns.**

3. Some necessary information, including the summary of embryos used during microinjection, should be provided.

**Response: A summary of embryos used for microinjection can now be found in Supplementary Table 1.**

4. Professional language editing is required.

**Response: Our manuscript was revised by a professional native English editor.**

**Reviewer #2:**

The authors provided answers to the previous questions. However, response to Comment 3 needs to be complemented by incorporating a null variant of CMP to nCas9 and nCas9-RTase module. CMP has an stimulatory effect on nickase activity only or a synergistic effect on the RTase activity?

The effect of CMP on PE activity may be interpreted from the two aspects: One is from the increased nickase activity, and the other from improved downstream process including RT and ligation. I meant by the previous claim that the incorporated CMP may have a positive effect on RT via changes in overall structure of the PE module. However, the authors considered the CMP effects only with respect to increased nickase activity. Yes, CMP possibly improves nickase activity. But, could you rule out the possibility that CMP may favore the RT process via the structural fitness effects? This address can only be made with the incorporation of a null mutant of CMP to nCas9 and nCas9-RTase (PE) and comparison of the results.

**Response: Thank you for your comment. CMP-PE-V1 used a Gly-rich linker between HN1 and nCas9 (linker: TGSG); between nCas9 and H1G (linker: LEGGGGS); and between H1G and RTase (linker: SGGSSGGSSGSETPGTSESATPESSGGSSGGSS) to prevent changes in protein structure and function (Additional file 1: Table S6). Gly-rich linkers in particular are flexible, linking multiple domains in a single protein without affecting the function of each domain (Reddy Chichili VP. et al. 2013 Protein Sci.: Linkers in the structural biology of protein-protein interactions).**

**In a previous experiment, we analyzed whether CMP could alter the chromatin state of *Igf2*, a closed chromatin target. As a result, we identified that CMP-PE gradually changed the closed chromatin structure to an open one; in contrast, PE made no change to the chromatin structure. These findings are direct evidence that using CMP-PE improves editing efficiency by unwinding chromatin structures and increasing chromatin accessibility. Therefore, we suggest that regardless of the activity of nCas9 or RTase, using CMP-PE improves editing efficiency.**

**Following a reviewer’s comment, we also compared the scenario of nCas9 and nCas9-RTase (PE) having a null mutant of CMP. These data can be found below. We found that nCas9 without RTase only caused indels with low efficiency at the target site, and did not make the accurate desired mutations. Therefore, it was not possible to compare the activity of RTase in this experimental group.**
